# Selective deletion of PPARβ/δ in fibroblasts causes dermal fibrosis by attenuated LRG1 expression

**DOI:** 10.1038/s41421-018-0014-5

**Published:** 2018-04-03

**Authors:** Ming Keat Sng, Jeremy Soon Kiat Chan, Ziqiang Teo, Terri Phua, Eddie Han Pin Tan, Jonathan Wei Kiat Wee, Nikki Jun Ning Koh, Chek Kun Tan, Jia Peng Chen, Mintu Pal, Benny Meng Kiat Tong, Ya Lin Tnay, Xuan Rui Ng, Pengcheng Zhu, Shunsuke Chiba, Xiaomeng Wang, Walter Wahli, Nguan Soon Tan

**Affiliations:** 1 School of Biological Sciences, Nanyang Technological University, 60 Nanyang Drive, Singapore, 637551 Singapore; 2 Lee Kong Chian School of Medicine, Nanyang Technological University, Novena Campus, 11 Mandalay Road, Singapore, 308232 Singapore; 3 Department of Microbiology, Tumor and Cell Biology, Karolinska Institutet, Nobels väg 16, 17177 Stockholm, Sweden; 4 Biological Sciences and Technology Division, CSIR-North East Institute of Science and Technology, Jorhat, Assam 785006 India; 5 School of Physical and Mathematical Sciences, Nanyang Technological University, Singapore, 637371 Singapore; 6 Institute of Molecular and Cell Biology, Agency for Science Technology & Research, 61 Biopolis Drive, Proteos, Singapore, 138673 Singapore; 7 Department of Cell Biology, Institute of Ophthalmology, University College London, London, UK; 8 Singapore Eye Research Institute, Singapore, 169856 Singapore; 9 INRA ToxAlim, Chemin de Tournefeuille, Toulouse Cedex 3, UMR1331 France; 10 Center for Integrative Genomics, University of Lausanne, Le Genopode, Lausanne, Switzerland; 11 KK Research Centre, KK Women’s and Children Hospital, 100 Bukit Timah Road, Singapore, 229899 Singapore

## Abstract

Connective tissue diseases of the skin are characterized by excessive collagen deposition in the skin and internal organs. Fibroblasts play a pivotal role in the clinical presentation of these conditions. Nuclear receptor peroxisome-proliferator activated receptors (PPARs) are therapeutic targets for dermal fibrosis, but the contribution of the different PPAR subtypes are poorly understood. Particularly, the role of fibroblast PPARβ/δ in dermal fibrosis has not been elucidated. Thus, we generated a mouse strain with selective deletion of PPARβ/δ in the fibroblast (FSPCre-*Pparb/d*^−/−^) and interrogated its epidermal and dermal transcriptome profiles. We uncovered a downregulated gene, leucine-rich alpha-2-glycoprotein-1 (*Lrg1*), of previously unknown function in skin development and architecture. Our findings suggest that the regulation of *Lrg1* by PPARβ/δ in fibroblasts is an important signaling conduit integrating PPARβ/δ and TGFβ1-signaling networks in skin health and disease. Thus, the FSPCre-*Pparb/d*^−/−^ mouse model could serve as a novel tool in the current gunnery of animal models to better understand dermal fibrosis.

## Introduction

Connective skin diseases are commonly characterized by excessive deposition of collagen in the skin and internal organs^1,2^. The classical hallmark of collagen overproduction is caused by myofibroblasts that are undergoing a dysregulated balance of signaling mediators, including cytokines and growth factors, resulting in dermal fibrosis that is observed in such diseases^3^. A prototypical and well-studied example of a connective skin disease is scleroderma (SSc). To date, treatments for these conditions exist only to provide symptomatic reliefs and helps to prevent further complications^4^. The precise etiology for the various clinical manifestations, however, remains unclear. Many animal models have been developed to better understand the disease pathogenesis^5,6^. Although each animal model could not completely recapitulate all aspects of connective tissue diseases, these models collectively have advanced our understanding of the different aspects of these conditions. Importantly, they have underscored that connective tissue diseases, and the underlying dermal fibrosis and overproduction of collagen, are multigenic and involves communications among key cell types, such as keratinocytes, fibroblasts, and immune cells^5–7^.

Nuclear receptors (NRs) represent one of the largest classes of transcription factors that are also known to be druggable targets from the clinical perspective^8–10^. A variety of skin diseases is currently treated with FDA-approved drugs that target NRs, such as glucocorticoids (cortisone) and vitamin D (calcitriol)^11^. There is a total of 48 NRs in humans, yet only a handful of them have well-defined roles in dermal fibrosis and collagen overproduction, and thus are unexplored therapeutic targets for these conditions^12^. The discovery of peroxisome-proliferator activated receptors (PPARs) has attracted attention due to their crucial role in lipid homeostasis and tissue repair^8,11^. There are three PPAR subtypes: PPARα, β/δ, and γ. A recent study showed that pan PPAR agonist IVA337 was effective in the prevention and treatment of experimental skin fibrosis^13^. The study pointed to a role for PPAR in dermal fibrosis, however, the contribution of various PPAR subtypes remains unclear. The skin of whole-body PPARγ heterozygous and fibroblast-specific PPARγ-deficient mice did not exhibit any significant alterations in skin thickness or matrix accumulation. Nonetheless, the deletion of PPARγ resulted in enhanced susceptibility to bleomycin-induced skin fibrosis and specific agonists of PPARγ have been shown to alleviate the extent of the development of cutaneous sclerosis^14,15^. As the most dominant PPAR subtype in the skin, the role of epidermal PPARβ/δ in skin physiology is well-documented^16–19^. Sclerodermic keratinocytes can trigger an inflammatory response from the fibroblasts via secreted IL-1α^20,21^. Interestingly, fibroblast PPARβ/δ has been shown to curb excessive epidermal proliferation during wound healing via the production of secreted IL-1 receptor antagonist^22^. Several studies have suggested that cellular redox state may play a significant role in the progression of scleroderma fibrosis^23,24^. Fibroblasts obtained from the skin of patients with SSc showed increase in reactive oxygen species production when compared to normal fibroblasts^25^. Incidentally, Wang et. al. underscored a novel role of fibroblast PPARβ/δ in the modulation of oxidative stress in a diabetic wound microenvironment^26^. These findings suggested a possible role for fibroblast PPARβ/δ in dermal fibrosis. However, whole-body PPARβ/δ knockout mice do not exhibit any dermal fibrotic phenotype^8,27–29^, and this may be because of a tissue-specific role for fibroblast PPARβ/δ.

To understand the role of PPARβ/δ in fibrotic skin diseases, we generated a fibroblast-selective PPARβ/δ deleted mouse (FSPCre-*Pparb/d*^−/−^). FSPCre-*Pparb/d*^−/−^ mice exhibited profound morphological changes to the skin architecture at an early age, pronounced wound contraction and have altered gene expression profile that resembles fibroproliferative diseases.

## Results

### PPARβ/δ-deficient fibroblasts enhance epidermal hyperplasia and inflammation

**Fig. 1:**
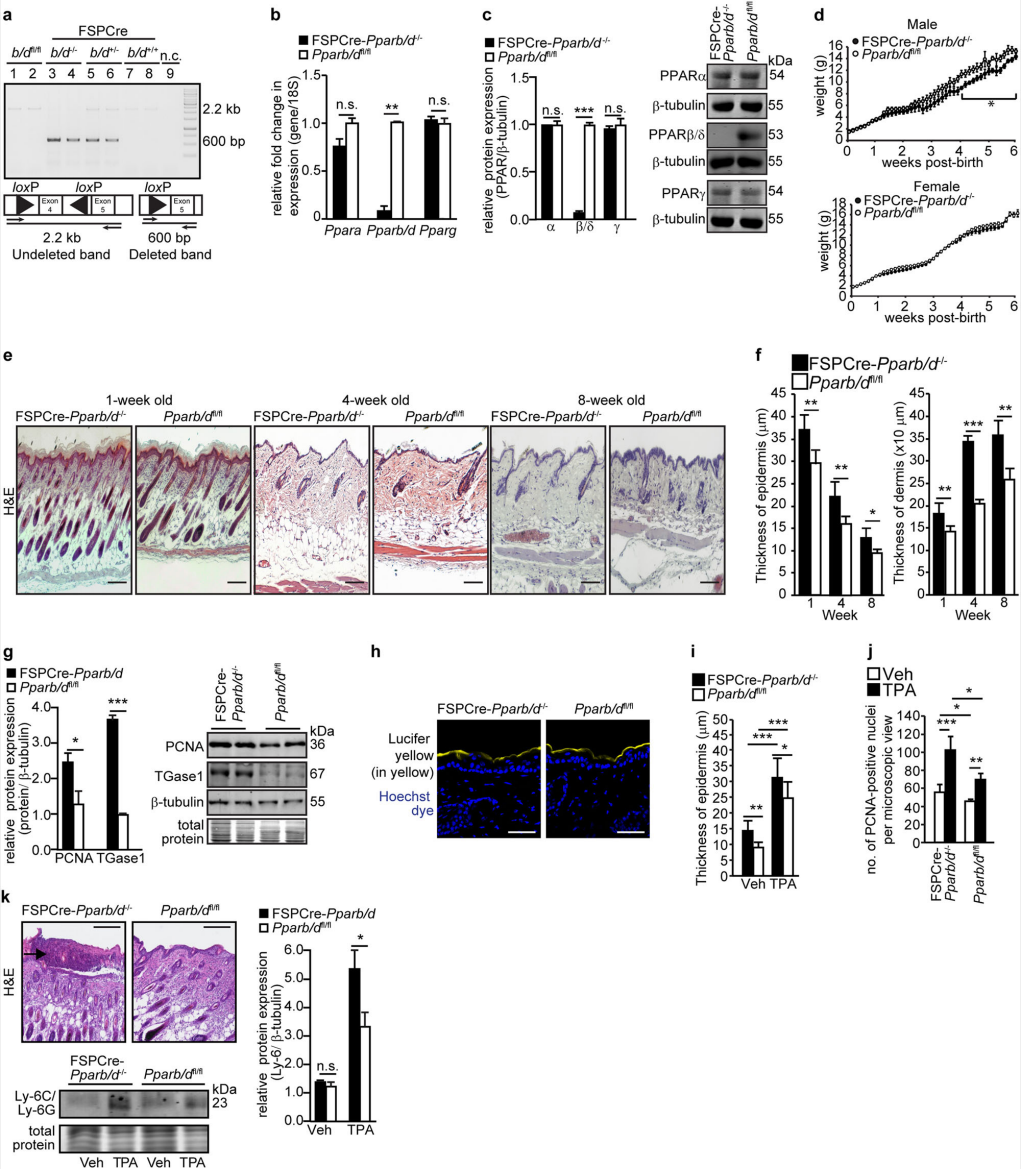
*Pparb/d* ablation in fibroblasts enhances epidermal inflammation. **a** PCR analysis of mouse fibroblast DNA. The non-deleted floxed allele is represented by the 2.2-kb band. The *Pparb/d*-deleted allele is represented by the 600-bp band. n.c., negative control. **b**, **c** Relative PPARα, PPARβ/δ, and PPARγ mRNA (**b**) and protein (**c**) levels in fibroblasts of FSPCre-*Pparb/d*^*fl/fl*^ and *Pparb/d*^*fl/fl*^ mice. Representative immunoblots are shown. 18S rRNA served as housekeeping gene. β-tubulin that served as housekeeping protein was from the same samples. Values are mean ± S.D. (*n* = 5). **d** Body weight of male and female mutant mice over 6 weeks post birth. Values represent mean ± S.D. (*n* = 35 for male and *n* = 40 for female mice). **e** Representative hematoxylin and eosin (H&E) stained skin sections from FSPCre-*Pparb/d*^*fl/fl*^ and *Pparb/d*^*fl/fl*^ mice at weeks 1, 4, and 8 of age. Scale bar = 50 μm. (*n* = 12). **f** Epidermis and dermis thickness in both genotypes at weeks 1, 4, and 8. Values represent mean ± S.D. (*n* = 31). **g** Relative protein expression of epidermal markers for proliferation (PCNA) and differentiation (TGase1) in FSPCre-*Pparb/d*^*fl/fl*^ and *Pparb/d*^*fl/fl*^ mice at age week 4. Representative immunoblots were shown. Coomassie stained of blots showed equal loading and transfer. β-tubulin that served as housekeeping protein was from the same samples. Values represent mean ± S.D. (*n* = 5). **h** Barrier function test. Representative images of skins from FSPCre-*Pparb/d*^*fl/fl*^ and *Pparb/d*^*fl/fl*^ mice topically treated with Lucifer yellow dye. Nuclei were stained with Hoechst dye (blue). Scale bar = 50 μm. **i**, **j** Graphs showing the epidermal thickness (**i**) and the number of PCNA-positive keratinocytes (**j**) on dorsal skin treated with either vehicle (Veh) or 6.5 nmol of 12-*O*-tetradecanoylphorbol-13-acetate (TPA) for 24 h. Values represent mean ± S.D. (*n* = 8). **k** Representative H&E stained section of TPA-treated skins from FSPCre-*Pparb/d*^*fl/fl*^ and *Pparb/d*^*fl/fl*^ mice. Intra-epidermal microabscesses were observed in skins from FSPCre-*Pparb/d*^*fl/fl*^ mice. Scale bar = 200 μm. Graph showing the relative protein expression of neutrophil marker Ly-6C/Ly-6G in Veh-treated and TPA-treated skin of FSPCre-*Pparb/d*^*fl/fl*^ compared to *Pparb/d*^*fl/fl*^ mice. Representative immunoblots are shown. Coomassie stained of blots show equal loading and transfer. Values represent mean ± S.D. (*n* = 5). *P < 0.05; **P < 0.01; ***P < 0.001; n.s. not significant

We generated a mouse whose exons 4–5 of PPARβ/δ gene were deleted in the fibroblasts by crossing *Pparb/d*^fl/fl^ with FSP1-Cre mice, herein called FSPCre-*Pparb/d*^−/−^. The excision of *Pparb/d*^fl/fl^ was identified by the amplification of a 600-bp DNA fragment in dermal fibroblasts isolated from FSPCre-*Pparb/d*^−/−^ and FSPCre-*Pparb/d*^+/-^ mice (Fig. 1a). Intact floxed *Pparb/d*^fl/fl^ appeared as a 2.2-kb band (Fig. 1a). Multiplex PCR of CD45^+^, CD31^+^, and MHC^+^ revealed differential deletion of PPARβ/δ allele in these isolated cells. No deleted PPARβ/δ allele was observed in either CD31^+^ or MHC^+^ cells, whereas PPARβ/δ was deleted in CD45^+^ cells (Fig. S1a). PPARβ/δ mRNA and protein were not detected in fibroblasts from FSPCre-*Pparb/d*^−/−^ mice (Fig. 1b, c). The relative expression of PPARα and PPARγ in FSPCre-*Pparb/d*^−/−^ fibroblasts was not altered when compared to that in *Pparb/d*^fl/fl^ fibroblast (Fig. 1b, c). This was similarly observed in human dermal fibroblasts whose endogenous PPARβ/δ was suppressed by siRNA (Fig. S1b). The deletion did not affect the number of pups per litter or gender ratio (Fig. S1c). The body weight of male offspring was significantly lower than that of their wildtype counterparts, but this difference was not observed in female offspring (Fig. 1d, S1d). Thus, the FSPCre-*Pparb/d*^−/−^ mouse is viable and PPARβ/δ expression in fibroblasts is not critical for embryonic development.

To assess whether the FSPCre-*Pparb/d*^−/−^ mouse exhibited post-natal alterations, we performed histomorphometric analysis of the skin. The FSPCre-*Pparb/d*^−/−^ mice exhibited a thicker epidermis, dermis, and hypodermis (Fig. 1e, f, S1e). Immunohistochemical staining and western blot analysis revealed an elevated expression of PCNA (proliferation marker) as well as transglutaminase type 1 (TGase1; differentiation marker), suggesting an increase in the number of keratinocytes that proceeded to differentiate (Fig. 1g, S1f–g). We did not detect differences in the penetration of Lucifer Yellow dye into the epidermis of FSPCre-*Pparb/d*^−/−^ and *Pparb/d*^fl/fl^ mice (Fig. 1h), suggesting that the skin barrier function was unaffected. Although, FSP1 has been reported to be a key marker of a specific subset of macrophages in the liver during fibrosis and injury^30^, the FSPCre-*Pparb/d*^−/−^ mice neither exhibited hepatomegaly nor had increased collagen deposition in the liver or gastrointestinal tract (Fig. S1h–i).

Previous studies showed that global PPARβ/δ knockout mice had an enhanced epidermal hyperplasia response when topically treated with 12-O-Tetradecanoylphorbol-13-acetate (TPA) compared to their cognate wild-type littermates^27,28^. We questioned the role of fibroblast PPARβ/δ in this acute TPA response, thus *FSPCre-Pparb/d*^*−/−*^ and *Pparb/d*^fl/fl^ mice were treated with TPA. We observed an enhanced epidermal hyperplasia with more PCNA-positive proliferating keratinocytes in FSPCre-*Pparb/d*^*−/−*^ mice compared to *Pparb/d*^*fl/fl*^ mice in response to topical TPA treatment (Fig. 1i, j, S1j–k). FSPCre-*Pparb/d*^*−/−*^ mice also exhibited intra-epidermal microabscesses with greater infiltration of Ly-6C/Ly-6G-positive neutrophils (Fig. 1k, S1l). Thus, PPARβ/δ ablation in fibroblasts led to an aggravated inflammatory response of the adjacent epidermis to TPA.

### FSPCre-*Pparb/d*^−/−^ mice exhibit dermal thickening and faster dermal wound healing

**Fig. 2:**
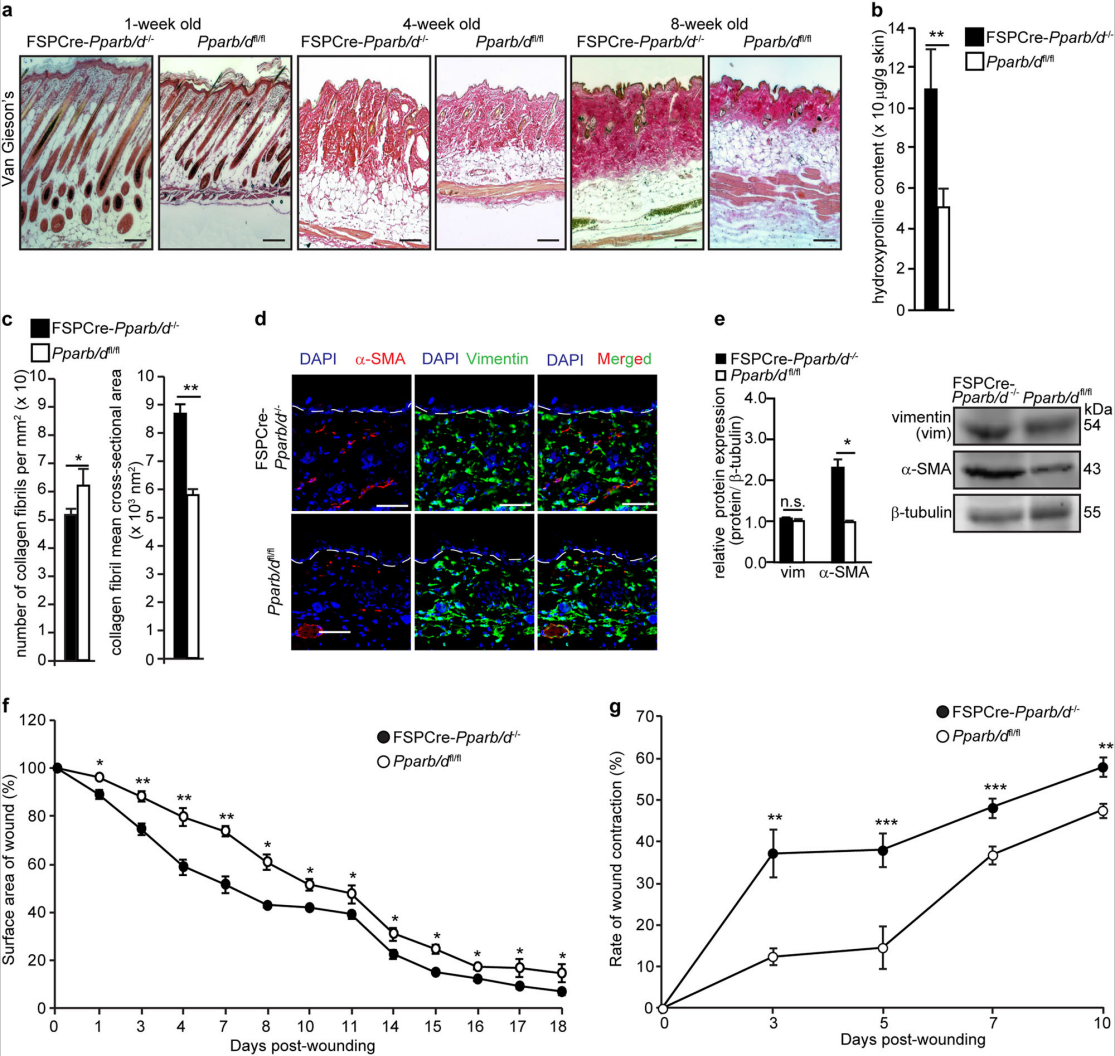
Increased collagen deposition in dermis of FSPCre-*Pparb/d*^*−/−*^ mice. **a** Representative Van Gieson’s stained sections of skins from FSPCre-*Pparb/d*^*−/−*^ and *Pparb/d*^*fl/fl*^ mice at weeks 1, 4, and 8. Scale bar = 50 μm. **b** Hydroxyproline content in FSPCre-*Pparb/d*^*−/−*^ and *Pparb/d*^*fl/fl*^ mice at week 4. Hydroxyproline content was normalized to total protein concentration. Values represent mean ± S.D. (*n* = 5). **c** Mean number and cross-sectional area of collagen fibrils in the dermis of the FSPCre-*Pparb/d*^*−/−*^ and *Pparb/d*^*fl/fl*^ mice at week 4. Values represent mean ± S.D. (*n* = 5). **d** Representative images of immunofluorescence staining for vimentin (green) and α-SMA (red) in dermis of both genotypes. Sections were counterstained with DAPI for nuclei (blue). The dotted line represents the epidermis-dermis junction. Scale bar = 50 μm. **e** Relative protein expression of vimentin (vim) and α-SMA in skin tissues from FSPCre- *Pparb/d*^*−/−*^ and *Pparb/d*^*fl/fl*^ mice. Representative immunoblots for vimentin and α-SMA are shown. β-tubulin served as housekeeping protein and was from the same samples. Values represent mean ± S.D. (*n* = 5). **f** Wound closure rate of full-thickness excisional wounds on FSPCre-*Pparb/d*^*−/−*^ and *Pparb/d*^*fl/fl*^ mice over a period of 18 days. Values are mean ± S.D. (Mann-Whitney U test; *n* = 7 per time point). **g** Rate of wound contraction determined as the distance between the first hair follicles on the wound edges. A 8-mm full-thickness excisional wound was created on dorsal backs of mice. Wound biopsies were harvested at the indicated days post-wounding and stained with H&E. Values are mean ± S.D. (Mann-Whitney U test; *n* = 10 per time point). *P < 0.05; **P < 0.01; ***P < 0.001; n.s. not significant

Dermal thickening was the most obvious phenotypic difference between FSPCre-*Pparb/d*^−/−^ and *Pparb/d*^fl/fl^ mice. There was more collagen deposition in the FSPCre-*Pparb/d*^−/−^ dermis compared with *Pparb/d*^fl/fl^ as indicated by Van Gieson’s stain and hydroxyproline measurement (Fig. 2a, b). We also stained the tissues using PicroSirus Red, which is intended for use in the histological visualization of Type I and III collagen in red and the cytoplasm in yellow. When viewed under polarized light microscope, a red fluorescence indicates the presence of Type 1 collagen (Fig. S2a). Consistently, we observed a thicker dermis and more collagen in the FSPCre-*Pparb/d*^−/−^ compared with *Pparb/d*^fl/fl^ mice (Fig. S2a). Focused ion beam scanning electron microscopy of the dermis region revealed a larger mean cross-sectional area but fewer collagen fibrils in FSPCre-*Pparb/d*^−/−^ mice compared to *Pparb/d*^fl/fl^ (Fig. 2c, S2b). We also detected more α-smooth muscle actin (α-SMA)-positive myofibroblasts, but not vimentin-positive fibroblasts in FSPCre-*Pparb/d*^−/−^ mice compared to *Pparb/d*^fl/fl^ mice (Fig. 2d, e). These findings suggest that PPARβ/δ deficiency in fibroblasts promotes the activation of fibroblasts to myofibroblasts, leading to increased dermal collagen deposition. To study how this phenotype affects dermal wound healing, we performed full-thickness excisional wounding assays. FSPCre-*Pparb/d*^−/−^ mice exhibited more rapid wound closure than the *Pparb/d*^fl/fl^ mice (Fig. 2f, S2c), which was associated with a significantly higher rate of wound contraction (Fig. 2g, S2d). These observations suggest that the higher number of myofibroblasts and increased collagen deposition with thicker collagen fibers contributed to the rapid wound contraction in FSPCre-*Pparb/d*^−/−^ mice.

### FSPCre-*Pparb/d*^−/−^ mice exhibit a pro-fibrotic gene expression profile

The above observations suggest that FSPCre-*Pparb/d*^−/−^ mice exhibited certain localized SSc-like hallmarks, including excessive collagen deposition leading to thickening of the dermis and greater number of myofibroblasts. Previous studies also concluded that localized SSc involves autoimmune abnormalities with elevated levels of anti-nucleosome antibodies^31,32^. We detected higher levels of anti-nucleosome antibodies in 69% of our FSPCre-*Pparb/d*^−/−^ mice, with a higher frequency in female mice than male mice (Fig. S2e). To further strengthen our findings, we performed comparative microarray gene expression analysis of skin biopsies from SSc patients, other mouse models of SSc and FSPCre-*Pparb/d*^−/−^ mice.

An intrinsic SSc gene set comprising 995 genes was previously identified by Milano et al (GSE9285)^33^. We examined the expression of these genes in the tight skin (Tsk) 1 or 2 heterozygous mutant mouse model (GSE71999), the bleomycin-induced fibrosis mouse model (GSE71999), the sclerodermatous graft versus host disease (SGVHD) mouse model (GSE24410), and our FSPCre-*Pparb/d*^−/−^ mice (Fig. S3). Unguided hierarchical clustering of samples revealed that the mouse models of SSc mainly represented the fibroproliferative subtypes of human SSc. Both fibroproliferative 1 and 2 subtypes of human SSc share a common biological program of proliferation^33–35^. FSPCre-*Pparb/d*^−/−^ mice samples branched off from the same node as fibroproliferative 2 subtype of human SSc samples, indicating that the gene expression profiles of the two sample groups are highly similar (Fig. S3).

**Fig. 3:**
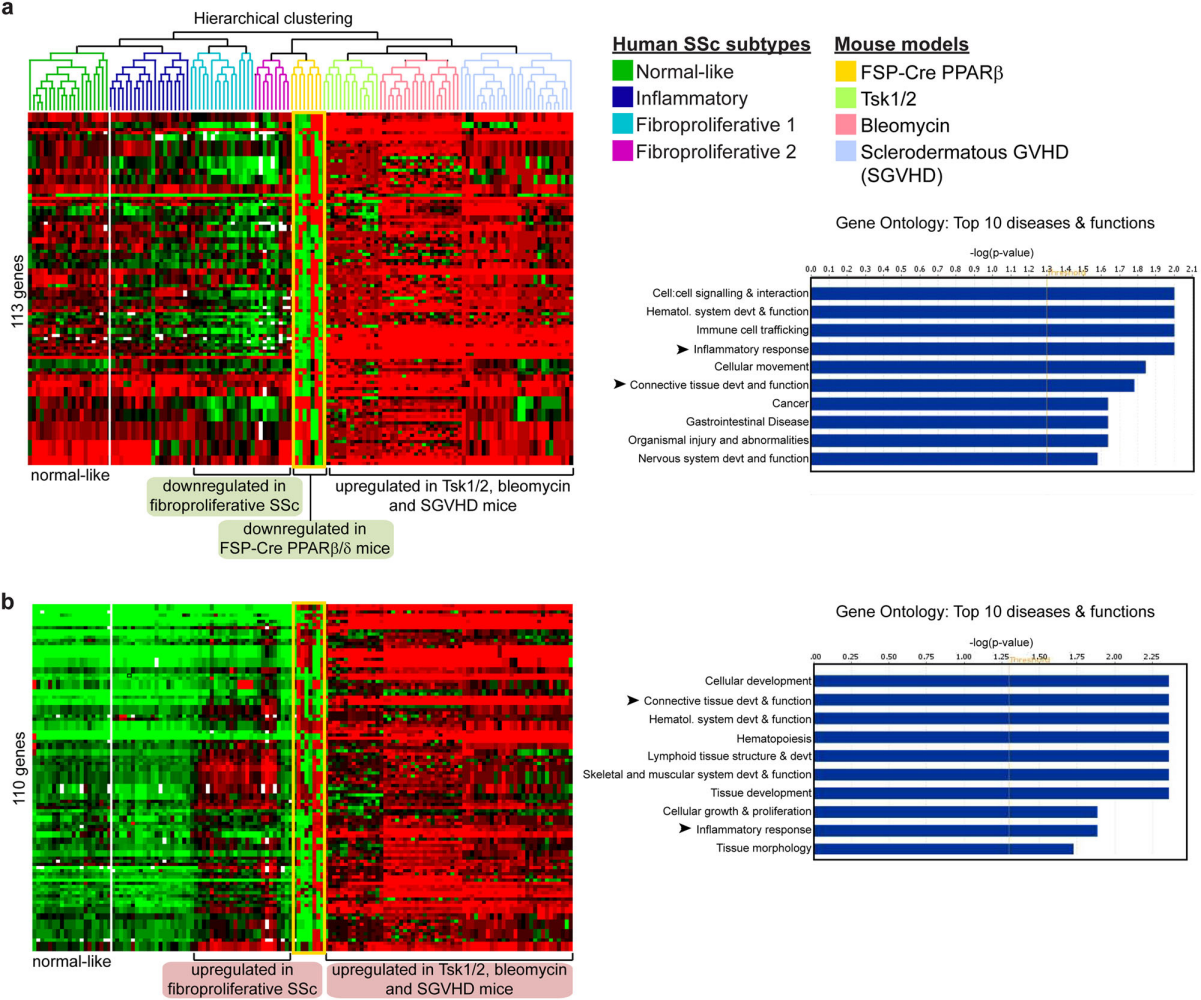
Mouse models of SSc recapitulate the fibroproliferative subset of human SSc. **a** Microarray datasets from FSPCre-*Pparb/d*^*−/−*^ mice were hierarchically clustered with microarray datasets from Tsk1/2 heterozygous mutant mice, bleomycin-induced fibrosis mice, SGVHD mice, and human SSc. Each mouse model or human disease cluster is represented by a unique color. Microarray datasets for other mouse models of SSc and human SSc were downloaded from the NCBI GEO database. FSPCre-*Pparb/d*^*−/−*^ mice (yellow box) uniquely recapitulated the expression pattern of 113 genes in human fibroproliferative SSc, i.e., 113 genes that were downregulated in human fibroproliferative SSc were similarly downregulated in FSPCre-*Pparb/d*^*−/−*^ mice. Bar charts represent the gene ontologies (GO) associated with the 113 gene set, ranked by –log(*P*-value). Arrows represent GOs that are highly relevant to SSc. **b** Tsk1/2 heterozygous mutant, bleomycin-induced fibrosis, bleomycin-induced fibrosis and SGVHD mice recapitulated the expression pattern of 110 genes in human fibroproliferative SSc, i.e., 110 genes that were upregulated in human fibroproliferative SSc were similarly upregulated in previous mouse models of scleroderma. Bar charts represent the gene ontologies (GO) associated with the 110-gene set, ranked by –log(*P*-value). Arrows represent GOs that are highly relevant to SSc

A subset of 113 genes involved in cell:cell signaling and interaction, connective tissue development and function, and inflammation was downregulated in fibroproliferative human SSc compared with normal human skin (Fig. 3a). On the one hand, only our FSPCre-*Pparb/d*^−/−^ mice recapitulated this molecular signature of the diseased tissue, whereas these 113 genes were mostly upregulated in the Tsk1/2, SGVHD and bleomycin-induced mouse models of SSc. On the other hand, a subset of 110 genes that was upregulated in human SSc samples was better represented by Tsk1/2, SGVHD and bleomycin-induced mouse models of SSc than FSPCre-*Pparb/d*^−/−^ mice (Fig. 3b). However, we noted that the gene ontologies (GO) of deregulated genes in all mouse models of SSc, including our FSPCre-*Pparb/d*^−/−^ mice, highlight connective tissue abnormalities and inflammation as critical processes in the development of SSc.

**Fig. 4:**
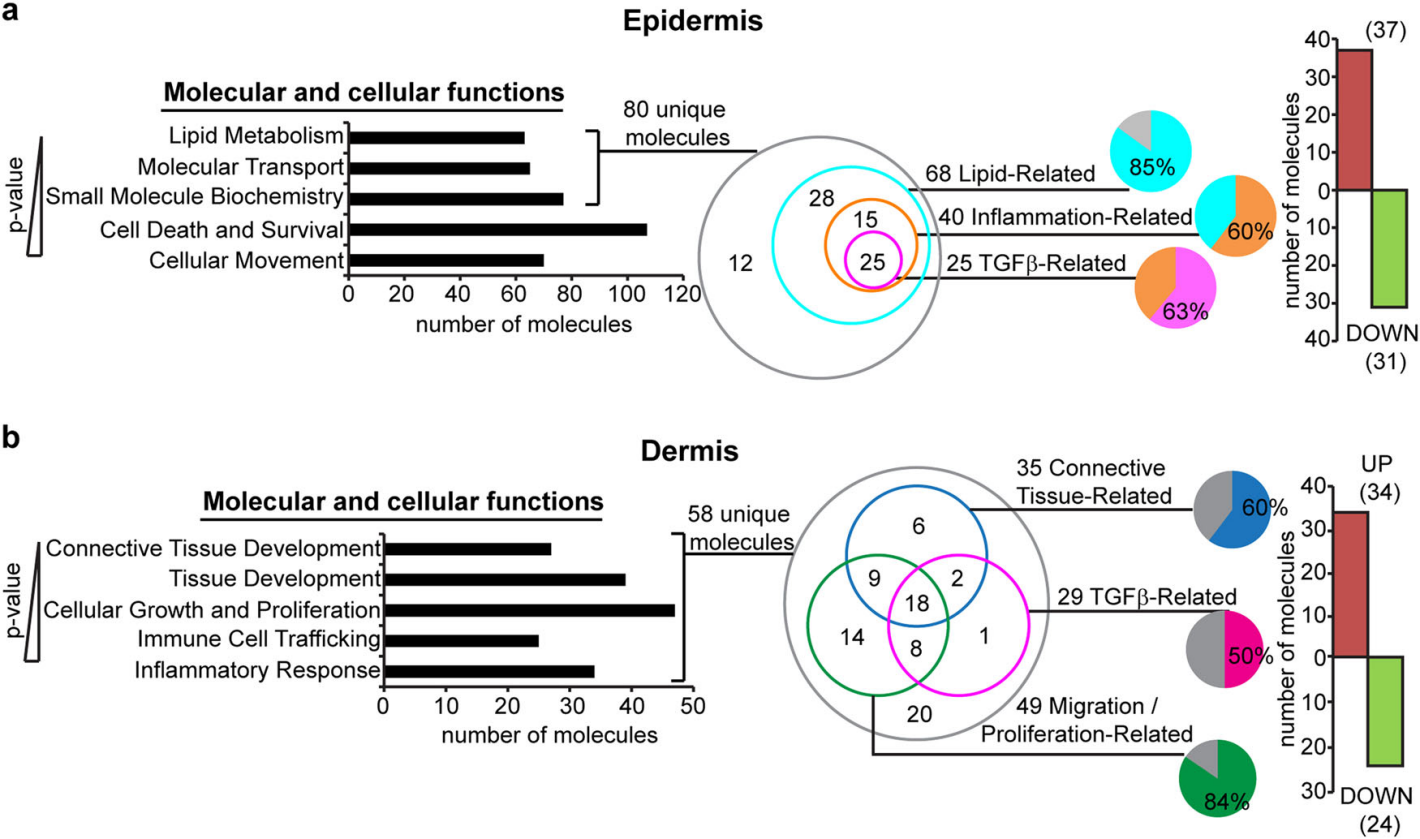
Effect of TGFβ1 signaling on skin architecture in FSPCre-*Pparb/d*^*−/−*^ mice. **a** Ingenuity pathway analysis (IPA) of genes differentially expressed by >1.5-fold in FSPCre-*Pparb/d*^*−/−*^ epidermis relative to *Pparb/d*^*fl/fl*^ epidermis. Top 5 gene ontologies (GO) (*P* < 0.0341) and their numbers of constituent molecules were identified. Lower *P*-values indicate stronger associations with the indicated GO. Further stratification of the 80 unique molecules identified lipid-related, inflammation-related, and TGFβ-related sub-ontologies is shown. The bar chart represents the number of genes upregulated (red) or downregulated (green) by >1.5-fold in FSPCre-*Pparb/d*^*−/−*^epidermis compared to *Pparb/d*^*fl/fl*^ epidermis. **b** IPA of genes differentially expressed by >1.5-fold in FSPCre-*Pparb/d*^*−/−*^dermis relative to *Pparb/d*^*fl/fl*^ dermis. Top 5 GO (*P* < 0.0197) and their numbers of constituent molecules were identified. Lower *P*-values indicate stronger associations with the indicated GO. Further stratification of the 58 unique molecules identified connective tissue-related, migration/proliferation-related, and TGFβ-related sub-ontologies. The bar chart represents the number of genes upregulated (red) or downregulated (green) by >1.5-fold in FSPCre-*Pparb/d*^*−/−*^dermis compared to *Pparb/d*^*fl/fl*^ dermis

Because our FSPCre-*Pparb/d*^−/−^ mice uniquely recapitulated the cell:cell signaling aspect of fibroproliferative 2 subtype of human SSc, we separated the epidermis and the dermis of FSPCre-*Pparb/d*^−/−^ mice skin to analyze their compartment-specific gene expression by microarray. Genes that were differentially regulated by >1.5-fold in the epidermis of FSPCre-*Pparb/d*^−/−^ mice enriched the GO of lipid metabolism, molecular transport, small molecule biochemistry, cell death and survival, and cellular movement, ranked according to their *P*-values (a lower *P*-value indicates a stronger association to the indicated GO) (Fig. 4a). A total of 80 unique gene transcripts were responsible for the top three enriched GOs in the epidermis. Among these 80 genes, 85% of them (68 genes: 37 upregulated, 31 downregulated) were lipid-related. 40 of these 68 genes (~60%) had overlapping functions in inflammation, and 25 of these 40 inflammation-related genes (~63%) were involved in TGFβ1 signaling (Fig. 4a, S4a). In the dermis, 58 genes that were differentially regulated by >1.5-fold, enriched the GOs of connective tissue development, tissue development, cellular growth and proliferation, immune cell trafficking and inflammatory response. 49 of the 58 genes (~85%) were migration/proliferation-related, 35 genes (~60%) were connective tissue-related and 29 genes (50%) were involved in TGFβ1 signaling (Fig. 4b, S4b).

The findings from microarray analysis of the two skin compartments suggest that TGFβ1 signaling is a major paracrine axis that is altered by the deletion of PPARβ/δ in fibroblasts. The microarray data is consistent with the increased immune cell infiltration within the skin of FSPCre-*Pparb/d*^−/−^ mice compared with their wild-type counterparts. This is also consistent with our current observation of epidermal hyperproliferation in the skin of FSPCre-*Pparb/d*^−/−^ mice. These observations suggest that the deletion of PPARβ/δ in fibroblasts creates a hotbed for facilitated immune cell entry, which is in part responsible for thickening of the skin.

### Fibroblast PPARβ/δ regulates *Lrg1* to modulate TGFβ1 signaling

**Fig. 5:**
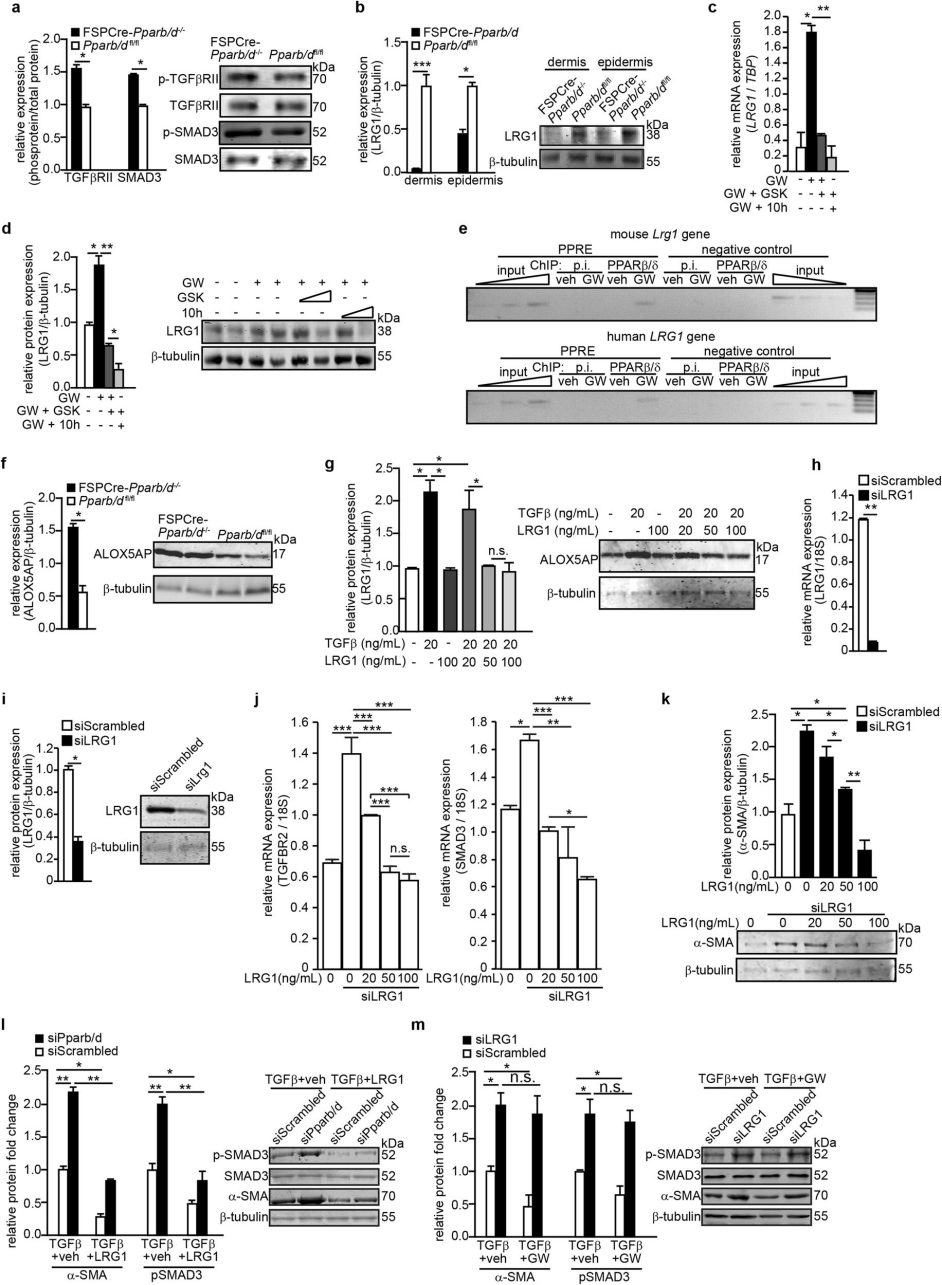
Fibroblast PPARβ/δ modulates TGFβ1 signaling via LRG1. **a**, **b** Relative expression levels of phosphorylated TGFβRII and SMAD3 (**a**) and LRG1 proteins in dermis and epidermis (**b**) from FSPCre-*Pparb/d*^*−/−*^and *Pparb/d*^*fl/fl*^ mice. Representative immunoblots for phosphorylated, total TGFβRII and SMAD3, as well as LRG1 were shown. β-tubulin served as housekeeping protein was from the same samples. Values are mean ± S.D. (*n* = 5). **c**, **d** Relative LRG1 mRNA (**c**) and protein (**d**) levels in human fibroblasts treated with GW501516 (GW), a specific PPARβ/δ agonist, and in combination with GSK0660 (GSK), a PPARβ/δ antagonist, and compound **10h** (**10h**), a PPARβ/δ inverse agonist. TBP, TATA-Box binding protein, served as housekeeping gene for qPCR. Representative immunoblots are shown. β-tubulin that served as housekeeping protein was from the same samples. Values are mean ± S.D. (*n* = 5). **e** Chromatin immunoprecipitation (ChIP) using PPARβ/δ antibodies or pre-immune Ig (p.i.) in human and mouse fibroblasts that were stimulated with either vehicle (Veh) or GW. Enrichment of either a PPRE-containing fragment from the endogenous *Lrg1* promoter or a control DNA fragment (2 kb upstream of the PPRE) was evaluated by PCR. Aliquots of the extract were also analyzed before immunoprecipitation (input). **f**, **g** Relative ALOX5AP protein level in skins from FSPCre-*Pparb/d*^*−/−*^and *Pparb/d*^*fl/fl*^ mice (**f**) and in human keratinocytes treated with either TGFβ1 or recombinant LRG1 proteins (**g**). Representative immunoblots for ALOX5AP were shown. β-tubulin served as housekeeping protein was from the same samples. Values represent mean ± S.D. (*n* = 5). **h**, **i** Relative LRG1 mRNA (**h**) and protein (**i**) levels in human fibroblasts transfected with either siRNA against LRG1 or scrambled siRNA control. 18 S rRNA served as housekeeping gene for qPCR. Representative immunoblot for LRG1 is shown. β-tubulin that served as housekeeping protein was from the same samples. Values are mean ± S.D. (*n* = 3). **j**, **k** Relative expression of TGFBR2, SMAD3 mRNAs (**j**) and α-SMA protein (**k**) in control, LRG1-knockdown human fibroblasts, and LRG1-knockdown human fibroblasts treated with an increasing dose of recombinant LRG1 protein. 18S rRNA served as housekeeping gene for qPCR. Representative immunoblot for α-SMA was shown. β-tubulin that served as housekeeping protein was from the same samples. Values are mean ± S.D. (*n* = 3). **l**, **m** Relative protein levels of α-SMA, SMAD3, and phosphorylated SMAD3 (p-SMAD3) in PPARβ/δ- (siPparb/d) (**l**), LRG1- (siLRG1) knockdown fibroblasts (**m**) and control fibroblasts transfected with scrambled siRNA (siScrambled). p-SMAD3 was normalized to total SMAD3 protein. β-tubulin that served as housekeeping protein was from the same samples. Values are mean ± S.D. (*n* = 3). *P < 0.05; **P < 0.01; ***P < 0.001; n.s. not significant

The interrogation of our microarray datasets suggested that dysregulated TGFβ1 signaling plays a central role in the profibrotic phenotype of FSPCre-*Pparb/d*^−/−^ mice (Fig. S4). Indeed, FSPCre-*Pparb/d*^−/−^ mice exhibited an enhanced activation of TGFβRII and SMAD3 via phosphorylation compared to *Pparb/d*^fl/fl^ mice (Fig. 5a). Of interest is the diminished expression of LRG1 in the dermis of FSPCre-*Pparb/d*^−/−^ mice (Fig. 5b, S4b). LRG1 modulates TGFβ1 signaling in a tissue-specific manner and has been implicated to play a role in vascularization^36,37^. Thus, the role of LRG1 is aligned with the dysfunctional microvascularization and dysregulated TGFβ1 signaling observed in SSc patients. PPARβ/δ agonist GW501516 (GW) increased LRG1 mRNA and protein levels, which were attenuated either by PPARβ/δ antagonist GSK0660 (GSK) or an inverse agonist compound **10h** (**10h**) in normal primary human fibroblasts (Fig. 5c, d). In silico analysis of the intragenic regulatory region of mouse and human *Lrg1* promoters revealed putative PPAR response elements (PPREs) (Fig. S5). Chromatin immunoprecipitation of primary mouse and human fibroblasts showed that PPARβ/δ is recruited to the PPRE of *Lrg1* (Fig. 5e). These experiments identified *Lrg1* as a direct target of PPARβ/δ in fibroblasts.

Microarray analyses revealed that the expression of genes related to lipid mediators of inflammation was upregulated in FSPCre-*Pparb/d*^−/−^ epidermis (Fig. 4a). One of the most upregulated genes was arachidonate 5-lipoxygenase-activating protein (*Alox5ap*), which mediates inflammatory responses towards external stimuli^38^ (Fig. S4). The FSPCre-*Pparb/d*^−/−^ mice expressed higher levels of ALOX5AP mRNA and protein in the epidermis than *Pparb/d*^*fl/fl*^ mice (Fig. 5f). Recombinant LRG1 decreased ALOX5AP expression in a dose-dependent manner in primary human keratinocytes (Fig. 5g). These results also suggest that the expression of ALOX5AP in the epidermis was regulated, in part, by paracrine production of LRG1 by the fibroblasts. We conclude that the upregulation of ALOX5AP in the epidermis of FSPCre-*Pparb/d*^−/−^ mice may contribute to the increased susceptibility of the epidermis to inflammatory insults.

The contractile and secretory α-SMA-positive myofibroblasts contribute to tissue repair during wound healing, but organ function can be severely impaired when contraction and collagen secretion become excessive, such as in fibrosis.^3,39^ We studied the TGFβ1-mediated α-SMA expression in LRG1-deficient fibroblasts (Fig. 5h, i). LRG1 deficiency led to an increase in the mRNA levels of TGFβRII and SMAD3, whereas treatment of LRG1-deficient fibroblasts with recombinant LRG1 reduced TGFβRII and SMAD3 expression (Fig. 5j). The increased expression of α-SMA observed in LRG1-deficient fibroblasts was indicative of enhanced fibroblast activation (Fig. 5k), consistent with the higher number of α-SMA-positive fibroblasts in FSPCre-*Pparb/d*^−/−^ mice. The exposure to increasing doses of recombinant LRG1 protein decreased the expression of α-SMA in a dose-dependent manner (Fig. 5k). To examine if recombinant LRG1 protein could rescue the fibrotic phenotype of PPARβ/δ-deficient fibroblasts, we treated control (siScrambled) and PPARβ/δ-knockdown (siPPARb/d) human fibroblasts with TGFβ1 in the presence or absence of recombinant LRG1 protein (Fig. 5l). TGFβ1 treatment resulted in higher expression of phosphorylated SMAD3 (p-SMAD3) and α-SMA in siPPARb/d fibroblasts when compared with siScrambled fibroblasts (Fig. 5l). Importantly, this increase expression of p-SMAD3 and α-SMA was attenuated in the presence of recombinant LRG1. Conversely, we treated siScrambled-knockdown and LRG1-knockdown (siLRG1) human fibroblasts with TGFβ1 in the presence or absence of PPARβ/δ agonist GW501516 (GW) (Fig. 5m). Similar to observation in Fig. 5k, reduced LRG1 resulted in higher p-SMAD3 and α-SMA, i.e., greater fibroblast activation in siLRG1 fibroblasts when compared with control. The co-treatment with GW did not significantly attenuate the increase (Fig. 5m). Thus, LRG1 acted downstream of PPARβ/δ and was able to rescue the fibrotic phenotype of PPARβ/δ-deficient fibroblasts.

These observations suggest a role of fibroblast PPARβ/δ in the homotypic regulation of TGFβ1 signaling, via increased LRG1 production, in fibroblast activation and collagen production, which contribute to the fibrotic phenotype of FSPCre-*Pparb/d*^*−/−*^ mice. Taken together, our results indicate that in fibroblasts, PPARβ/δ transcriptionally upregulates Lrg1 that in turn acts in an autocrine and paracrine manner to modulate tissue-specific TGFβ1 effects.

### Ligand-activated PPARβ/δ reduces the fibrotic effect in SSc

**Fig. 6:**
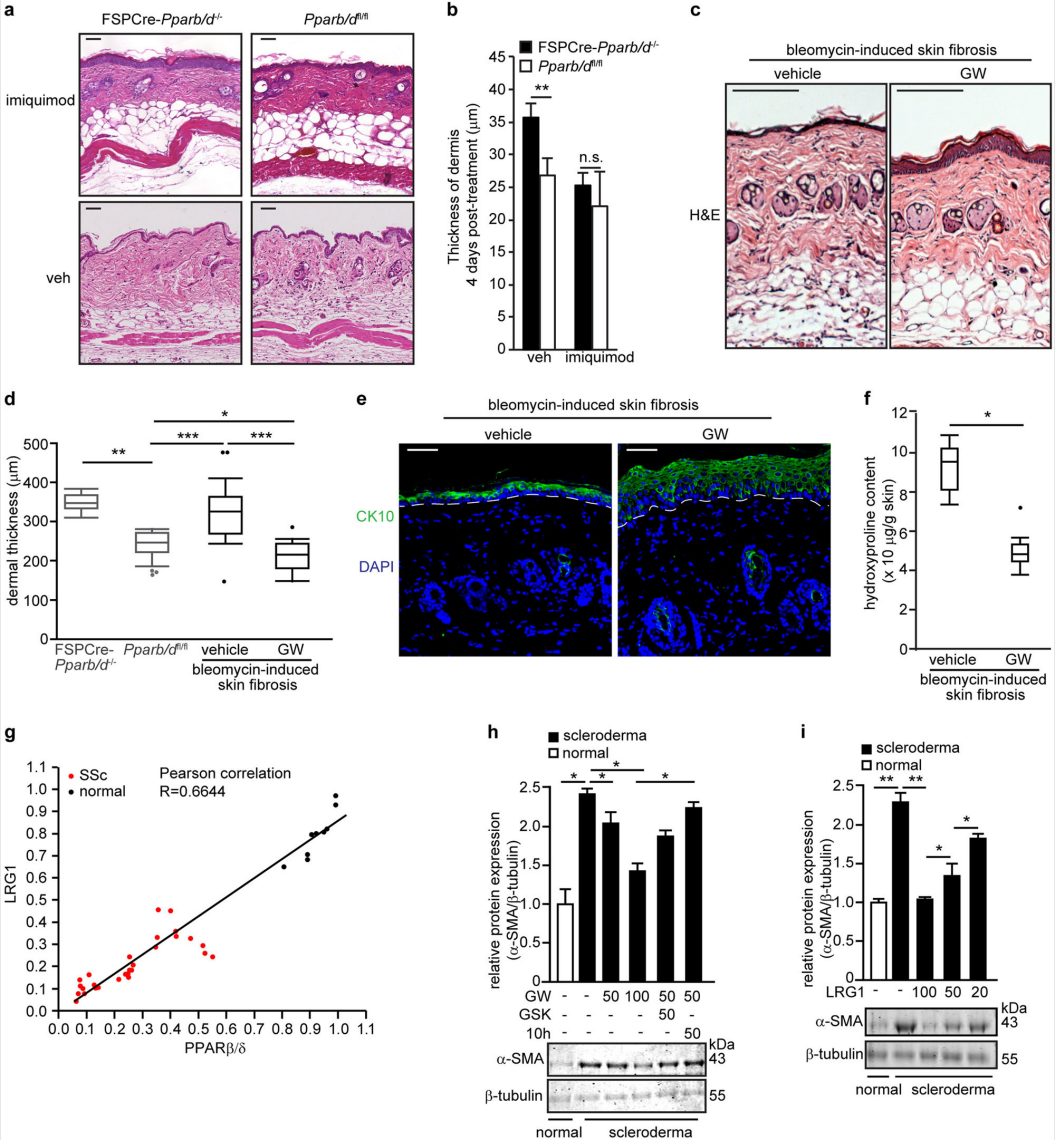
PPARβ/δ and LRG1 reduce bleomycin-induced dermal fibrosis. **a** Representative H&E stained sections of FSPCre-*Pparb/d*^*−/−*^and *Pparb/d*^*fl/fl*^ mice skin treated with imiquimod cream 4% daily for 4 days compared to vehicle-treated (Veh) cognate mice. Scale bar = 50 μm. **b** Graph showing the dermal thickness of Veh-treated and imiquimod-treated skins from indicated mice. Values represent mean ± S.D. (*n* = 5). **c** Representative H&E stained sections of vehicle-treated and GW-treated skins from bleomycin-induced SSc. Scale bar = 150 µm. **d** Dermal thickness of vehicle-treated and GW-treated skins from bleomycin-induced SSc. The dermal thickness of untreated FSPCre-*Pparb/d*^*−/−*^and *Pparb/d*^*fl/fl*^ mice skin was included for comparison purpose. Values represent mean ± S.D. (*n* = 5). **e** Representative images of immunofluorescence staining against cytokeratin 10 (CK10) in bleomycin-injected mouse skin that was treated with either vehicle or the PPARβ/δ agonist, GW. Scale bar = 50 µm. **f** Hydroxyproline content in vehicle-treated and GW-treated skins from bleomycin-induced sclerodermic mice. Hydroxyproline content was normalized to total protein concentration. Values represent mean ± S.D. (*n* = 5). **g** Correlation plot of PPARβ/δ and LRG1 proteins in fibroblasts from normal patients (*n* = 9) and from scleroderma patients (*n* = 28). Representative immunoblots for PPARβ/δ and Lrg1 are in Supplementary Figure S5b. **h**, **i** Relative expression of α-SMA protein in normal and sclerodermic fibroblasts treated with either PPARβ/δ agonist (GW), antagonist (GSK), and inverse agonist (**10h**) (**h**) or treated with increasing doses of recombinant LRG1 protein (**i**). Representative immunoblots for α-SMA are shown. β-tubulin that served as housekeeping protein was from the same samples. Values are mean ± S.D. (*n* = 3). *P < 0.05; **P < 0.01; ***P < 0.001; n.s. not significant

Although the best treatment for localized SSc is uncertain and current treatments often fail to help, benefits have been reported following topical 5% imiquimod cream (Aldara®) treatment^40–43^. Imiquimod is an immune response modifier. It induces interferon and subsequently inhibits TGFβ1 and was reported to attenuate SSc symptoms in patients^40^. FSPCre-*Pparb/d*^−/−^ mice topically treated with imiquimod exhibited a thinning of the thickened dermis (Fig. 6a). The resultant dermal thickness was no longer significantly different from *Pparb/d*^*fl/fl*^ (Fig. 6b). To evaluate the potential of PPARβ/δ as a therapeutic target for dermal fibrosis, we examined the effect of the PPARβ/δ agonist GW on the widely used bleomycin-induced mouse model of dermal fibrosis. Dermal fibrosis was induced in C3H/He wildtype mice through daily subcutaneous injection of bleomycin for a period of 28 days. We concurrently co-injected them with either dimethyl sulfoxide (DMSO) (vehicle) or GW. Histological analysis revealed that the dermis of the mice co-treated with GW were thinner than that of mice co-treated with vehicle (Fig. 6c, d). The epidermis of mice co-treated with GW showed increased differentiation of the epidermal layer by PPARβ/δ activation, as evidenced by an increased CK10 staining (Fig. 6e). Hydroxyproline assay also showed a lower level of collagen in the dermis of GW-treated mice (Fig. 6f).

To further underscore the clinical relevance of PPARβ/δ, we examined the effect of GW on human dermal fibroblasts that were explanted from 28 SSc patients. Scleroderma fibroblasts expressed lower levels of PPARβ/δ mRNA and protein than normal fibroblasts (Fig. 6g, h). The protein level of LRG1 was lower in SSc fibroblasts than in normal fibroblasts isolated from healthy volunteers (Fig. 6h). GW-activated PPARβ/δ reduced the expression of α-SMA in SSc fibroblasts, while the co-treatment with either GSK or compound **10h** (PPARβ/δ inverse agonist) increased the expression of α-SMA (Fig. 6i). Recombinant LRG1 similarly reduced the expression of α-SMA in SSc fibroblasts (Fig. 6j), indicating that PPARβ/δ exerted its effect through LRG1, a direct downstream target of PPARβ/δ. This observation suggested that the activation of PPARβ/δ could reduce fibrosis, underscoring its therapeutic potential.

## Discussion

Connective skin diseases are characterized by dermal fibrosis and excessive collagen deposition in the skin and internal organs. Fibroblasts are the main producers of collagen in the extracellular matrix, and thus the pivotal cell type involved in the clinical presentation of connective skin diseases, including SSc. Yet, the transcriptional networks within the fibroblasts that contribute to dermal fibrosis are not well-studied. We showed here that fibroblast PPARβ/δ is not critical for embryonic development in mice. However, the epidermis of FSPCre-*Pparb/d*^*−/−*^ mice had an aggravated response to TPA, suggesting that PPARβ/δ ablation in the fibroblasts rendered the mutant mice more susceptible to developing inflammatory skin disorders. Notably, FSPCre-*Pparb/d*^−/−^ mice exhibited pronounced dermal thickening associated with excessive collagen production, a greater number of myofibroblasts and Ly6-positive immune cells, which somewhat represents a fibrosing skin disorder phenotype. These observations were also supported by our comparative gene analyses, which showed that the expression of the respective genes for collagen production and immune cells activation were correspondingly altered.

The FSPCre-*Pparb/d*^−/−^ mice exhibited dermal fibrosis. The skin of FSPCre-*Pparb/d*^−/−^ mice was characterized by increased collagen synthesis associated with increased activation of fibroblasts to myofibroblasts. However, FSPCre-*Pparb/d*^−/−^ livers exhibited neither hepatomegaly, which manifests with systemic SSc-associated primary biliary cirrhosis^44^, nor increased collagen deposition in the intestinal wall, which could cause bowel hypomobility^45^. These indicate that the role of PPARβ/δ in fibroblasts is likely to be tissue/organ-specific. We detected dysregulated TGFβ1 signaling in the FSPCre-*Pparb/d*^−/−^ mice, which has been implicated in the etiology of dermal fibrosis^46^. Dermal fibrotic symptoms may be further aggravated by an enhanced epidermal inflammatory response in these mutant mice. In clinical settings, activated fibroblasts give rise to an immune-inflammatory environment with abnormal repair of damaged vessels and fibrosis^47,48^ and ROS production^49–52^. PPARβ/δ-deficient fibroblasts contribute to greater microenvironment oxidative stress due to reduced expression of antioxidant genes such as catalase and glutathione peroxidase 1^26^. Microarray analyses of FSPCre-*Pparb/d*^−/−^ skin revealed upregulation of numerous genes that would culminate an increased ROS production in the PPARβ/δ-ablated dermis, including CCL2 and CCL5. Notably, CCL2 and CCL5 upregulation was found to precede skin and lung fibrosis in a mouse model that recapitulates the pathology of SSc^53^. Finally, we also identified gene networks in FSPCre-*Pparb/d*^*−/−*^ mice that closely resemble fibroproliferative 2 subtype of human SSc. Thus, the fibroblast PPARβ/δ deficiency causes dermal fibrosis. Unlike FSPCre-*Pparb/d*^*−/−*^ mice, fibroblast-specific PPARγ-deficient skin did not exhibit any manifestations of dermal fibrosis or collagen overproduction, suggesting that fibroblast PPARβ/δ and PPARγ play different roles in skin physiology^14^. FSPCre-*Pparb/d*^*−/−*^ exhibited epidermal hyperplasia which is characteristic of psoriasis, another type of connective tissue diseases. Several studies have also implicated a role for PPARβ/δ in psoriasis^54–58^. However, the relative contribution of epidermal and dermal PPARβ/δ in the etiology and pathophysiology of psoriasis remains unclear. This study is still ongoing in the lab, and is beyond the scope of this work.

Our current study revealed that TGFβ1 signaling has an overarching influence on skin architecture in FSPCre-*Pparb/d*^−/−^ mice. We identified *Lrg1* as a direct downstream target of PPARβ/δ that contributes to the observed phenotype. Thus far, LRG1 had been reported to augment endothelial TGFβ1 signaling and promote angiogenesis^36^. LRG1 is expressed in various human tissues, including the breast, skin, and intestine, but little is known about its role in these tissues. Our study shows that fibroblast-secreted LRG1 acts in a paracrine manner to influence the epidermal TGFβ1 response to inflammatory challenges. The underlying mechanism involved transcriptional upregulation of LRG1 by fibroblast PPARβ/δ, which modulated homotypic and heterotypic TGFβ1 signaling. Limitations of our study include that one genetic background of mouse and our deletion strategy consists of the deletion of exons coding for the DNA-binding domain of PPARβ/δ. It is also conceivable that other gene deletion strategies may result in different phenotypic severity or outcomes^8^. In addition to fibroblasts, FSP1 driven gene deletion may affect other cell types, although in vitro experiments were performed to strengthen the conclusion. Nevertheless, to the best of our knowledge, this is the first study to examine the role of fibroblast PPARβ/δ in skin, which reveals novel epithelial–mesenchymal communication.

Current treatment of SSc is directed toward managing complications and providing symptomatic relief. Although several regimens have shown benefit in case series, few controlled trials have been performed and data suggest wide variation in the approach to treatment^59^. For example, topical 5% imiquimod cream has been reported for the treatment of localized SSc^40^. We showed that FSPCre-*Pparb/d*^−/−^ mice were responsive to topical treatment with imiquimod cream. The dermis of these mice became thinner, resulting in a thickness similar to that of their wildtype littermates. We also showed that ligand-activated PPARβ/δ reduced dermal fibrosis using the bleomycin-induced mouse model of dermal fibrosis. In the 28 SSc patients analyzed, fibroblasts consistently had reduced level of PPARβ/δ and LRG1 compared to healthy subjects. In addition, PPARβ/δ activation, or LRG1 treatment, could reduce α-SMA levels in these profibrotic fibroblasts. In conclusion, we identified an important signaling conduit that integrates PPARβ/δ and TGFβ1 signaling networks via LRG1 to be key players in fibrosis.

## Materials and methods

### Cell culture

Human keratinocytes (Lonza, USA) were routinely cultured in keratinocyte-SFM culture media (Invitrogen, USA). Fibroblasts were maintained in FibroGRO^TM^ Medium (Millipore, USA). All cultures were maintained in a humidified incubator at 37 ^o^C with 5% CO_2_. Normal human dermal and sclerodermic fibroblasts were procured from Asterand Bioscience, UK and Proteogenex, USA. Scleroderma patients were classified accordingly to American College of Rheumatology criteria^60^. SSc patients presented limited cutaneous SSc with skin thickening was limited to hands and face. SSc patients have Raynaud’s phenomenon for >5 years, minimal joint pain and presented telangiectasias. The male:female ratio was approximately 2:7. The balanced median age of the normal (*n* = 9) and SSc (*n* = 28) donors were 42.8 ± 3.1 and 49.1 ± 2.5 years old, respectively. Due to the role of PPARβ/δ in energy homeostasis, patients with diabetes, obese patients (BMI > 30) or patients with hyperlipemia were excluded. Skin fibroblasts were isolated from skin biopsy of the forearm using the explant method^61^ and flash frozen at the third passages for subsequent analysis.

### Antibodies and chemicals

Primary antibodies used in this study: monoclonal anti-α-SMA, PCNA, GAPDH, polyclonal anti-PPARβ/δ, β-tubulin, TGase1, collagen type I and ALOX5AP (Santa Cruz, USA); polyclonal anti-Ly6C/Ly-6G (BioLegend, USA); polyclonal anti-vimentin and LRG1 (Abcam, USA). IRDye^®^ 680LT conjugated secondary antibodies were from Li-Cor (Biosciences, USA); DyLight^®^ conjugated secondary antibodies were from Vector Laboratories, USA. GW501516 (PPARβ/δ agonist; GW), GSK0660 (PPARβ/δ antagonist; GSK) and compound **10h** (PPARβ/δ inverse agonist; **10h**) were synthesized in-house.

### Generation of fibroblast-selective PPARβ/δ-knockout mice

The *PPARb/d*^*fl/fl*^ mouse was provided by Prof Walter Wahli (University of Lausanne, Switzerland). The *PPARb/d*^*fl/fl*^ mouse carries alleles of the *PPARβ/δ* gene where the exon 4, which encodes the DNA-binding domain, was flanked by loxP sites^62,63^. The FSPCre transgenic mouse expresses Cre recombinase under the control of the FSP1 promoter^64^. FSPCre mice were always maintained as hemizygotes. FSPCre and *PPARb/d*^*fl/fl*^ mice were independently backcrossed to C57BL/6J wild-type mice for at least six generations. The FSPCre-*Pparb/d*^+/-^ mouse was generated in-house by crossing *PPARb/d*^*fl/fl*^ and FSPCre mice. FSPCre-*Pparb/d*^+/-^ progenies were bred with *Pparb/d*^fl/fl^ mice to obtain FSPCre-*Pparb/d*^−/−^ mice and the wild-type control mice. To sustain this genotype, we bred FSPCre-*Pparb/d*^−/−^ progenies with *Pparb/d*^fl/fl^ mice. Mice were housed in a specific-pathogen free facility with a 12 h/12 h light/dark cycle. Food and water were provided *ad libitum*.

Mice in the telogen phase of their hair cycle were anaesthetized and depilated on their dorsal back before being subjected to a biopsy punch (~8 mm). The skin biopsy was then treated with 3.8% ammonium thiocyanate (Sigma, USA) in 1 × PBS for 30 min at 37 °C. Subsequently, the epidermis was mechanically separated from the dermis with a pair of forceps. The dermis was then curetted with a 30-gauge needle. The dermis was washed with 1 × PBS before being subjected to the genotyping procedure, real-time qPCR assay and Western blot. Mice genotyping were performed using the Mouse Genotyping Kit HotStart as described by manufacturer (KAPA Biosystems, USA). Genotyping primers are provided in Table S1.

Animal experiments were carried out in accordance to the guidelines of the University Institutional Animal Care and Use Committee (IACUC, ARF-SBS/NIE-A0216AZ, A0321, and A0322), Singapore.

### Real-time qPCR and Western blot

RNA extraction, reverse transcription, qPCR and western blot were performed as previously described^65^. Total RNA was extracted using TRIzol® as described by manufacturer (Invitrogen, USA). Reverse transcription was performed using the iScript^TM^ Reverse Transcription Supermix for RT-qPCR (Bio-Rad, USA) and qPCR done using KAPA SYBR® FAST Universal qPCR Kit (KAPA Biosystems, USA). Primer sequences are provided in Table S1. Total protein was extracted using M-PER™ Mammalian Protein Extraction Reagent (ThermoFisher Scientific). Infrared western blot was done as previously described^66^. Protein bands were analyzed using the Odyssey CLx scanner and Image Studio V2.1 (LI-COR Biosciences, USA).

### Histological, immunohistochemical, and immunofluorescence staining

Tissues were processed, sectioned and stained as previously described^65^. PicroSirius Red staining was performed as recommended by the manufacturer (ab150681, Abcam, Cambridge, United Kingdom). Colorimetric images were captured using Axio Scan.Z1 with Plan Apochromat 20×/0.8 objective (Carl Zeiss, Germany). Fluorescence images were captured using the LSM 710 confocal microscope with the Zeiss EC Plan-NEOFLUAR 20×/0.5 NA objective. Analyses were performed with ZEN 2012 Light Edition software (Carl Zeiss, Germany).

### *Lucifer* yellow permeability assay

Lucifer yellow dye (1 mg/mL; Sigma-Aldrich, USA) was topically applied on the epidermis for 1 h. Excess Lucifer yellow was washed away with 1 × PBS, and the skin tissues were cryofrozen with OCT tissue freezing medium (Leica, USA). Tissues were cryosectioned at 8 μm and mounted with Hoechst 33,342 dye (Life Technologies, USA). Images were taken using the LSM 710 confocal microscope with the Zeiss EC Plan-NEOFLUAR 20×/0.5 NA objective. Analyses were performed with ZEN 2012 Light Edition software (Carl Zeiss, Germany).

### Hydroxyproline assay

Hydroxyproline content in tissues was determined as previously described^67^. Trans-4-hydroxy-L-proline (0–300 µg/mL) was used to generate a standard graph. Absorbance was measured at 550 nm using a SpectraMax M2e Multi-Mode Microplate Reader and SoftMax Pro Microplate Data Acquisition & Analysis Software (Molecular Devices, USA). The value of unknown hydroxyproline was determined from the standard graph. The weight of each respective wound biopsy was used for normalization.

### 12-O-tetradecanoylphorbol 13-acetate (TPA)-induced skin inflammation

Mice of ~4 weeks of age were shaven on their dorsal skin and topically treated with 6.5 nmol of TPA (Sigma-Aldrich, USA) in acetone for 24 h. The vehicle-treated and TPA-treated skins were then harvested and processed accordingly for histological studies and protein analysis.

### Wounding experiment

Mice of ~8 weeks of age were shaved and full-thickness excisional wounds were inflicted on the dorsal skin of mice as previous described^68^. Wound surface areas and contraction rates were measured over the indicated duration.

### Imiquimod topical application

Mice of ~8 weeks of age were shaved and subsequently imiquimod cream 4% (Selleck Chemicals, USA) was applied topically daily for a period of 4 days. Skin tissues were then harvested and processed for histological analysis.

### Bleomycin-induced dermal fibrosis

C3H/He mice of 4 weeks of age were subcutaneously injected with 10 µg of bleomycin sulfate (Selleck Chemicals, USA) together with either DMSO (*n* = 5) or 0.25 mg of GW (*n* = 5) daily for a period 28 days^69^. Skin tissues were then harvested and processed accordingly for histological studies.

### Gene expression microarray

cDNA synthesis was performed using the Applause WT-Amp ST System (NuGEN Technologies, USA). The resulting cDNA was purified with MinElute Reaction Cleanup Kit (Qiagen, USA), followed by fragmentation and labeling using the Encore Biotin Module (NuGEN Technologies, USA). The fragmented and labeled cDNA were mixed with a hybridization master mix (Affymetrix, USA) in accordance to the manufacturer’s protocol, and 90 μL of the resultant mixture were injected into the GeneChip® Mouse Gene 1.0 ST Array gene chips (Affymetrix, USA). The scanned data was collected for analyses using the Partek Genomics Suite v6.6 (Partek Inc., USA). Differences in gene expression between FSPCre-*Pparb/d*^*−/−*^ and wild-type samples were identified using the ANOVA function of the Partek Genomics Suite. Microarray datasets from FSPCre-*Pparb/d*^*−/−*^ mice were subsequently hierarchically clustered with microarray datasets from tight skin (Tsk) 1 or 2 heterozygous mutant mice (GSE71999), bleomycin-induced fibrosis mice (GSE71999), sclerodermatous graft versus host disease (SGVHD) mice (GSE24410), and human samples of SSc (PMC2481301, GSE9285), downloaded from NCBI Gene Expression Omnibus (GEO) database. We focused our analysis on an intrinsic SSc gene set comprising 995 genes that was previously reported^33^ (GSE9285). Gene ontology (GO) analysis of gene subsets was performed using the Ingenuity Pathway Analysis software (Ingenuity Systems Inc., USA). The microarray protocols and data have been deposited in NCBI’s Gene Expression Omnibus (GEO) database accession number GSE71419.

### Chromatin immunoprecipitation (ChIP)

ChIP experiments were performed as previously described^65^ except that anti-PPARβ/δ antibodies was used (see above). ChIP primer sequences are listed in Table S1.

### Transfection and luciferase assay

TGFβ1 responsive luciferase reporter assay in keratinocytes was performed as previously described^70^. Luciferase assay was performed using the Dual Luciferase® Reporter Assay System and the Glomax 20/20 Luminometer (Promega, USA). The ON-TARGETplus SMARTpool siRNA were used for knockdown studies (Lrg1, Cat # L-056177-01; PPARβ/δ, Cat #L-042751-01; Dharmacon). Cells were transfected using DharmaFect 1 as recommended by manufacturer. As control, the ON-TARGETplus non-targeting control pool (D-001810-10; Dharmacon) was used.

### Anti-nucleosome antibody (ANA) detection

Mice of 4–6 weeks of age were studied for their ANA levels. ANA levels in mice plasma samples were measured by using the anti nucleosome IgG Antibody (AnuA, IgG) BioAssay^TM^ ELISA Kit (United States Biological, USA). Readings were performed on the Infinite® M200 Pro machine (Tecan, Switzerland) with the Magellan^TM^ v7.0 software (Tecan, Switzerland).

### Statistical analysis

Statistical analyses were performed using two-tailed Mann-Whitney U test using SPSS software (IBM Corporation, USA). A *P*-value of <0.05 was considered significant.

## Electronic supplementary material

Supplemental Information

## Electronic supplementary material

**Supplementary Information** accompanies the paper at (10.1038/s41421-018-0014-5).
