# Supplementary material for: Selective deletion of PPARβ/δ in fibroblasts causes dermal fibrosis by attenuated LRG1 expression
Source: Cell Discov. 2018 Apr 3;4:15. doi: 10.1038/s41421-018-0014-5 (PMC5880809; doi:10.1038/s41421-018-0014-5)

Selective deletion of PPARβ/δ in fibroblasts causes dermal fibrosis by attenuated LRG1 expression.

Ming Keat SNG[1,3], Jeremy Soon Kiat CHAN[1], Ziqiang TEO[1], Terri PHUA[1,2], Eddie Han Pin TAN[1], Jonathan Wei Kiat WEE[1], Nikki Jun Ning KOH[1], Chek Kun TAN[3], Jia Peng CHEN[3], Mintu PAL[4], Benny Meng Kiat TONG[5], Ya Lin TNAY[5], Xuan Rui NG[3], Pengcheng ZHU[1], Shunsuke CHIBA[5], Xiaomeng WANG[3,6,7,8], Walter WAHLI[3,9,10] and Nguan Soon TAN[1,3,6,11]

[1]School of Biological Sciences, Nanyang Technological University, 60 Nanyang Drive, Singapore 637551.
[2]Karolinska Institutet, Department of Microbiology, Tumor and Cell Biology, Nobels väg 16, Stockholm 17177, Sweden.
[3]Lee Kong Chian School of Medicine, Nanyang Technological University, Novena Campus, 11 Mandalay Road, Singapore 308232.
[4]Biological Sciences and Technology Division, CSIR-North East Institute of Science and Technology, Jorhat, Assam 785006, India
[5]School of Physical and Mathematical Sciences, Nanyang Technological University, Singapore 637371.
[6]Institute of Molecular and Cell Biology, 61 Biopolis Drive, Proteos, Agency for Science Technology & Research, Singapore 138673.
[7]Department of Cell Biology, Institute of Ophthalmology, University College London, United Kingdom.
[8]Singapore Eye Research Institute, Singapore 169856.
[9]INRA ToxAlim, UMR1331, Chemin de Tournefeuille, Toulouse Cedex 3, France.
[10]Center for Integrative Genomics, University of Lausanne, Le Genopode, Lausanne, Switzerland.
[11]KK Research Centre, KK Women's and Children Hospital, 100 Bukit Timah Road, Singapore 229899.

Correspondences to:
MKS: Email: mksng@ntu.edu.sg
NST (Senior Corresponding Author): Email: nstan@ntu.edu.sg
Tel: +65 6316 2941; Fax: +65 6791 3856

Supplementary Figure Legends

Figure S1. FSPCre-*Pparb/d*$^{-/-}$ mice are viable and have thicker epidermis and dermis.

(a) Representative scatterplots and gel runs of CD45^{+} (left panel), CD31^{+} and MHC^{+} positive cells (right panel) from indicated tissues of FSPCre-*Pparb/d*$^{-/-}$and *Pparb/d*$^{fl/fl}$ mice. The diagram showed the relative position of the multiplex genotyping primers, resulting in a larger deleted PPARβ/δ allele compared to floxed PPARβ/δ allele type.

(b) Relative PPARα, PPARβ/δ, and PPARγ mRNA and protein levels in human fibroblasts transfected with either control (siScrambled) or PPARβ/δ (siPPARβ/δ) siRNAs. Representative immunoblots are shown. 18S rRNA served as housekeeping gene. β-tubulin that served as housekeeping protein was from the same samples. Values are mean ± S.D. (n=3).

(c) Average number of male versus female pups in each litter. n.s., not significant.

(d) Body length of male and female mutant mice over 6-week post birth. Values represent mean ± S.D. (n=35 for male and n=40 for female mice).

(e) Thickness of the hypodermis and skeletal muscle for both genotypes at weeks 1, 4, and 8. Values represent mean ± S.D. (n=10).

(f-g) Representative sections of colorimetric staining of PCNA (d) and TGase1 (e) in the skin tissues of FSPCre-*Pparb/d*$^{-/-}$and *Pparb/d*$^{fl/fl}$ mice. Scale bar = 50 μm.

(h) Representative macroscopic images of liver from FSPCre-*Pparb/d*$^{-/-}$ and *Pparb/d*$^{fl/fl}$ mice.

(i) Representative immunoblots of type I collagen and β-actin from gastrointestinal tract and liver of FSPCre-*Pparb/d*$^{-/-}$ and *Pparb/d*$^{fl/fl}$ mice.

(j-l) Representative sections of H&E (j), immunohistochemical staining of PCNA (k) and Ly-6C/Ly-6G (l) for vehicle- (Veh) and TPA-treated skins of FSPCre-*Pparb/d*$^{-/-}$ and *Pparb/d*$^{fl/fl}$ mice. Scale bar = 50 μm. *$P < 0.05$; **$P < 0.01$; ***$P < 0.001$; n.s., not significant.

Figure S2. FSPCre-*Pparb/d*$^{-/-}$ mice show greater wound contraction.

(a) Representative PicroSiruis Red staining against collagen in the skins of FSPCre-*Pparb/d*$^{-/-}$ and *Pparb/d*$^{fl/fl}$ mice at 1 and 8 weeks of age (n=3 for each age). Scale bar = 50 µm.

(b) Representative transmission electron microscopy images of dermis of FSPCre-*Pparb/d*$^{-/-}$ and *Pparb/d*$^{fl/fl}$ mice. Scale bars = 0.2 µm (inset) and 1 µm.

(c) Macroscopic images of wounds from FSPCre-*Pparb/d*$^{-/-}$ and *Pparb/d*$^{fl/fl}$ mice at indicated day post-injury.

(d) Representative images of wound contraction at indicated day post-injury. Wound contraction is determined using the distance between the first hair follicles at the wound edges. Red arrows indicate wound edges. Scale bar = 500 µm.

(e) Frequency of male and female FSPCre-*Pparb/d*$^{-/-}$ mice that were positive for anti-nucleosome antibodies in their plasma. Seven male and 22 female mice were studied.

Figure S3. Microarray analyses of an intrinsic scleroderma gene set comprising 995 genes in mouse models of scleroderma and human SSc samples. Microarray datasets from FSPCre-*Pparb/d*$^{-/-}$ mice were hierarchically clustered with microarray datasets from Tsk1/2 heterozygous mutant mice, bleomycin-induced fibrosis mice, SGVHD mice, and human SSc. Each mouse model or human disease cluster is represented by a unique color. Microarray datasets for other mouse models of scleroderma and human SSc were downloaded from the NCBI GEO database. The white box represents a subset of 110 genes that are upregulated in human fibroproliferative SSc and well-represented by Tsk1/2 heterozygous mutant mice, bleomycin-induced fibrosis mice and SGVHD mice. The black box represents a subset of 113 genes that are downregulated in human fibroproliferative SSc and well-represented by FSPCre-*Pparb/d*$^{-/-}$ mice. Bar charts represent the gene ontologies (GO) associated with the 995 gene set, ranked by –log(p-value).

Figure S4. Gene ontology (GO) analyses of epidermis and dermis.

(a) GO of genes altered in the epidermis of FSPCre-*Pparb/d*$^{-/-}$ mice. Genes that were altered were highly associated with lipid metabolism (80 genes). Out of these 80 genes, approximately 60% (40 genes) were associated with inflammatory responses. One of the most up-regulated genes was arachidonate 5-lipoxygenase activating protein (*Alox5ap*; circled in red), with an up-regulation of approximately 4.6-fold.

(b) GO of genes altered in the dermis of FSPCre-*Pparb/d*$^{-/-}$ mice. Altered genes were highly associated with cellular function and maintenance, which could alter the behavior and function of fibroblasts in the dermis. Leucine-rich a-2-glycoprotein 1 (*Lrg1*) was one of the most down-regulated genes detected upon fibroblast-specific deletion of *Pparb/d*. This down-regulation was approximately 4-fold.

Figure S5. *Lrg1* is a novel direct target of PPARβ/δ.

(a) Schematic diagram showing the relative position of PPRE sequences in mouse and human *Lrg1* (red boxes) and their respective NUBIScan scores.

(b) Representative immunoblots of PPARβ/δ and Lrg1 from fibroblasts of normal (n = 9) and sclerodermic patients (n = 28). GAPDH served as housekeeping protein and was from the same samples. Densitometric values were used for the correlation plot.

Supplementary Table

Supplementary Table S1. Primer sequences used in this study (5' to 3').

**Mouse Genotyping**	
PPARβ/δ Exons 4/5 Forward	GCA GCT GCT CAG CTG CCT GC
PPARβ/δ Exon 4 Reverse	CCG CCT CTC GCC ATC CTT TCA GA
PPARβ/δ Exon 5 Reverse	CAG GTG CTG GGC GCC CTC TGA CAG AAT
FSP1Cre Control Forward	CTA GGC CAC AGA ATT GAA AGA TC
FSP1Cre Control Reverse	GTA GGT GGA AAT TCT AGC ATC ATC C
FSP1Cre Transgene Forward	GCG GTC TGG CAG TAA AAA CTA TC
FSP1Cre Transgene Reverse	GTG AAA CAG CAT TGC TGT CAC TT
**Real-time qPCR**	
Mouse PPARα Forward	TCG GCG AAC TAT TCG GCT G
Mouse PPARα Reverse	GCA CTT GTG AAA ACG GCA GT
Mouse PPARβ/δ Forward	TTG AGC CCA AGT TCG AGT TTG
Mouse PPARβ/δ Reverse	CGG TCT CCA CAC AGA ATG ATG
Mouse PPARγ Forward	TGT GGG GAT AAA GCA TCA GGC
Mouse PPARγ Reverse	CCG GCA GTT AAG ATC ACA CCT AT
Human Lrg1 Forward	CAG ACA GCG ACC AAA AAG C
Human Lrg1 Reverse	GGA ACA CCT GGC AGT CTT TG
Human Tgfbr2 Forward	CGT GGA GTC GTT CAA GCA GAC G
Human Tgfbr2 Reverse	CCG CAC CTT GGA ACC AAA TGG
Human Smad3 Forward	GAA CGT CAA CAC CAA GTG CAT
Human Smad3 Reverse	ACG CAG ACC TCG TCC TTC T
Human Col1A2 Forward	GGT CTC GGT GGG AAC TTT G
Human Col1A2 Reverse	CCA GGT GGG CCT CTA GGT
Rpl27 Forward	CGC AAA GCT GTC ATC GTG

Rpl27 Reverse	GTC ACT TTG CGG GGG TAG
18S Forward	GTA ACC CGT TGA ACC CCA TT
18S Reverse	CCA TCC AAT CGG TAG TAG CG
TBP Forward	GCT GGT TAT CGG GAG TTG G
TBP Reverse	ACT GGC CTG GTG TCC TAG AG
**Chromatin immunoprecipitation (ChIP)**	
Human Lrg1 Forward	CCA GGA ATA GTG CCT TGC AAA C
Human Lrg1 Reverse	CCA GTC CAG GCA GGT ATA AGG C
Human Lrg1 Negative Forward	CTC CTG GGT GCA AGC AAT TCT C
Human Lrg1 Negative Reverse	GGG ATT ACA GGC GTG AGC CA
Mouse Lrg1 Forward	ACC TGC TTG GTT CCT TGT GGA AGA C
Mouse Lrg1 Reverse	CAA ACT CAG TCC CTG GAT CCT GGT G
Mouse Lrg1 Negative Forward	TGT GCG TGT GTG CGA CTC AG
Mouse Lrg1 Negative Reverse	CTT CGA CCA CCA GCT GAC CAT

Synthesis of PPARβ/δ ligands

^{1}H NMR (400 MHz) spectra were recorded on a Bruker Avance 400 spectrometer in $CDCl_3$ [with $(CH_3)_4Si$ (δ = 0.00 ppm) as an internal standard]. ^{13}C NMR (100 MHz) spectra were recorded on a Bruker Avance 400 spectrometer in $CDCl_3$ [with residual $CHCl_3$ (d = 77.00 ppm) as an internal standard]. The following abbreviations are used to explain the multiplicities: s = singlet, d = doublet, t = triplet, q = quartet, sept = septet, br s = broad singlet, m = multiplet. High-resolution mass spectra were obtained with a Finnigan MAT 95 XP mass spectrometer (Thermo Electron Corporation). Flash column chromatography was performed by using Merck silica gel 60 with distilled solvents.

Synthesis of GW501516 (GW)

GW501516 (IUPAC: {4-[({4-methyl-2-[4-(trifluoromethyl)phenyl]-1,3-thiazol-5-yl}methyl)sulfanyl]-2-methylphenoxy}acetic acid) was prepared according to reported synthetic route as shown in Scheme 1 (Ham and Kang, 2005).

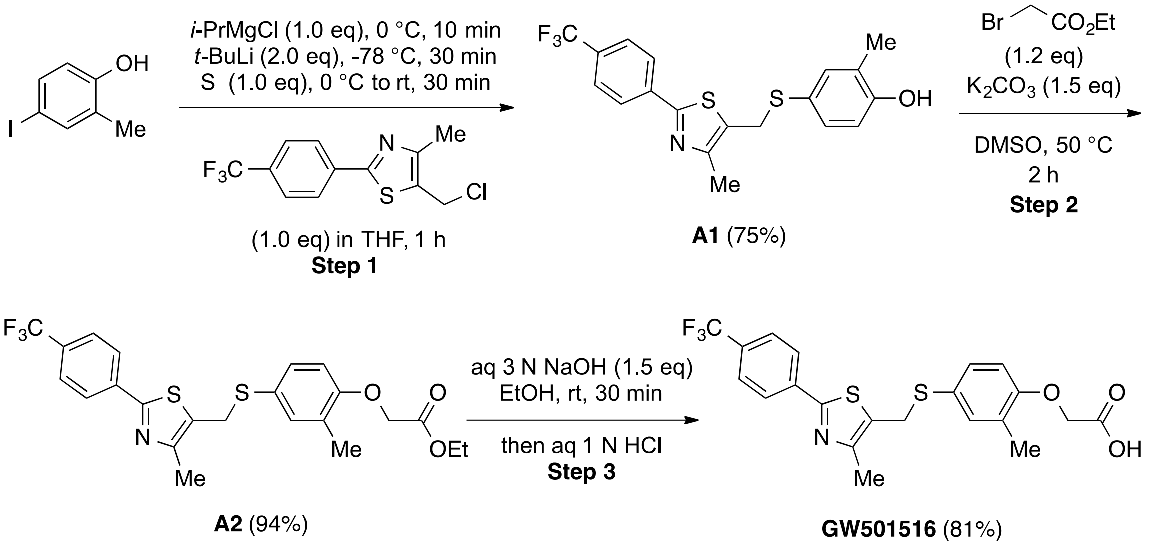

Scheme 1

Step 1: To a solution of 4-iodo-2-methylphenol (2.46 g, 10.5 mmol) in THF (80 mL) was added isopropylmagnesium chloride (5.3 mL, 2.0 M solution in THF, 10.5 mmol) at 0 °C under a N_2

atmosphere (Edgar and Falling, 1990). The mixture was stirred for 10 min at 0 °C, and then further cooled to –78 °C before *tert*-butyllithium (12.4 mL, 1.7 M solution in pentane, 21.0 mmol) was added dropwise and the reaction mixture was stirred for 30 min at –78 °C. A suspension of sulfur (337 mg, 10.5 mmol) in anhydrous THF (10 mL) was then dropped into the reaction mixture and then warmed to room temperature and left to be stirred for 30 min. Finally, the reaction mixture was cooled to 0 °C and a solution of 5-(chloromethyl)-4-methyl-2-(4-(trifluoromethyl)phenyl)thiazole (3.06 g, 10.5 mmol) in anhydrous THF (10 mL) was added dropwise, and then the reaction mixture was stirred at room temperature for 1 h (Sznaidman et al., 2003). The reaction was quenched with saturated aqueous NH_4Cl and then acidified with aqueous 1 M HCl. The mixture was extracted thrice with ethyl acetate and the combined organic extract was washed with brine and dried over anhydrous $MgSO_4$, filtered, and evaporated under reduced pressure to give the crude product. The crude compound was purified by silica-gel flash column chromatography (*n*-hexane/ethyl acetate=75:25) to obtain 2-methyl-4-(((4-methyl-2-(4-(trifluoromethyl)phenyl)thiazol-5-yl)methyl)thio)phenol (**A1**) (3.13 g, 7.9 mmol, 75%) as a white solid.

Step 2: To a solution of **A1** (2.87 g, 7.24 mmol) in DMSO/water (54 mL/ 6 mL) was added K_2CO_3 (1.49, 10.9 mmol) and ethyl bromoacetate (0.96 mL, 8.69 mmol) at room temperature. The reaction mixture was warmed to 50 °C and then vigorously stirred for 2 h. After the reaction was completed, the mixture was poured into water (100 mL) and extracted thrice with ethyl acetate. The combined organic extract was washed twice with water, dried over anhydrous $MgSO_4$, filtered, and evaporated under reduced pressure to give the crude product. The crude product was purified by silica-gel flash column chromatography (*n*-hexane/ethyl acetate=80:20) to obtain ethyl 2-(2-methyl-4-(((4-methyl-2-(4-(trifluoromethyl)phenyl)thiazol-5-yl)methyl)thio)phenoxy)acetate (**A2**) (3.26 g, 6.77 mmol, 94%) as a pale yellow solid. NMR analysis was in agreement with the literature data.

Step 3: A 3N aqueous solution of NaOH (3.40 mL, 10.2 mmol) was dropped into a solution of **A2** (3.26 g, 6.77 mmol) in ethanol (135 mL) and left to be stirred at room temperature for 30 min. Next, the reaction mixture was acidified with 1N HCl to pH 2–3 and ethanol was removed under reduced pressure. The residue was diluted with water and extracted thrice with ethyl acetate. The combined organic extract was then washed with brine, dried over anhydrous $MgSO_4$, filtered, and evaporated under reduced pressure to give the crude product. The crude compound was purified by silica-gel flash column chromatography (AcOH/ethyl acetate=1:99) to obtain GW501516 as a white solid (2.50 g, 5.51 mmol, 81%).

4-[({4-methyl-2-[4-(trifluoromethyl)phenyl]-1,3-thiazol-5-yl}methyl)sulfanyl]-2-methylphenoxy}acetic acid (GW501516)

^{1}H NMR (400 MHz, $CDCl_3$) δ 2.15 (s, 3H), 2.23 (s, 3H), 4.10 (s, 2H), 4.67 (s, 2H), 6.62 (d, 1H, *J* = 8.4 Hz), 6.82 (br s, 1H), 7.11 (dd, 1H, *J* = 8.4, 2.0 Hz), 7.22 (d, 1H, *J* = 1.6 Hz), 7.66 (d, 2H, *J* = 8.4 Hz), 7.95 (d, 2H, *J* =8.4 Hz). ^{13}C NMR (100 MHz, $CDCl_3$) δ 14.5, 16.1, 32.4, 65.1, 111.5, 123.9 (q, *J* = 271.0 Hz), 125.3, 125.9 (q, *J* = 4.0 Hz), 126.5, 128.4, 130.9, 131.6 (q, *J* = 33.0 Hz), 132.3, 136.3, 136.4, 151.2, 156.2, 163.7, 172.5.

Synthesis of GSK0660 (GSK)

GSK0660 (IUPAC: methyl 3-({[2-(methoxy)-4 phenyl]amino}sulfonyl)-2-thiophenecarboxylate) was synthesized using the synthetic route as shown in Scheme 2.

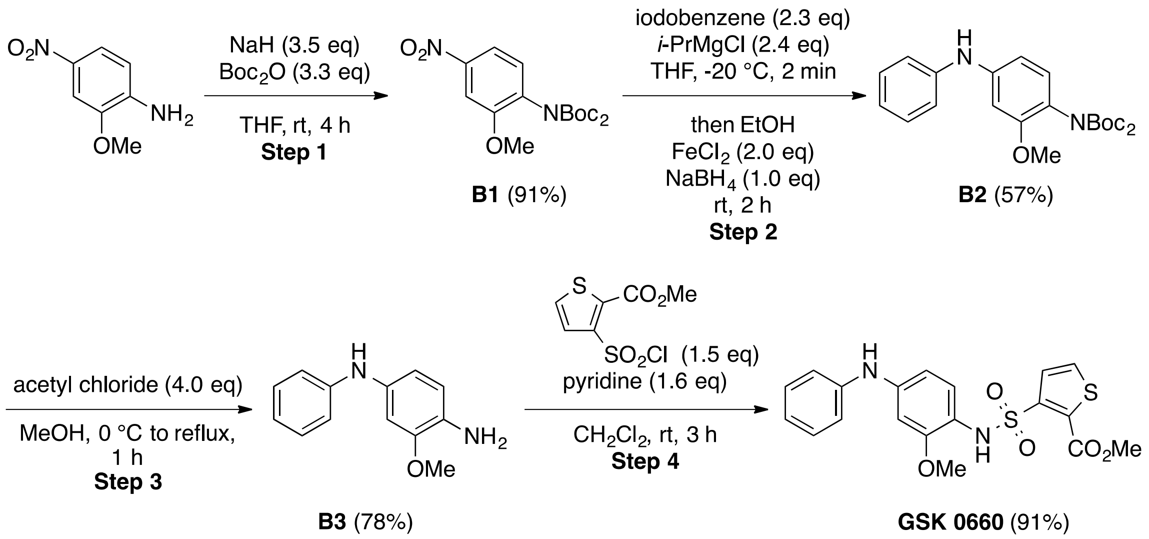

Scheme 2

Step 1: To a solution of 2-methoxy-4-nitroaniline (1.75 g, 10.4 mmol), NaH (1.46g, 60 % in mineral oil w/w, 36.4 mmol) in THF (100 ml) was added di-*tert*-butyl dicarbonate (7.49 g, 34.3 mmol). The mixture was stirred at room temperature under a N_2 atmosphere for 4 h to form a slurry solution. The reaction was quenched carefully by slow addition of water, and extracted thrice by ethyl acetate. The combined organic extract was washed with brine, dried over anhydrous $MgSO_4$, filtered, and evaporated under reduced pressure to give the crude product. The crude product was purified by silica-gel flash column chromatography (*n*-hexane/ethyl acetate=80:20) to obtain the *N*-protected 2-methoxy-4-nitroaniline (**B1**) (3.49, 9.47 mmol, 91%) as a pale yellow solid.

^{1}H NMR (400 MHz, $CDCl_3$) δ 1.41 (s, 18H), 3.94 (s, 3H), 7.27 (d, 1H, *J* = 8.5 Hz), 7.78 (d, 1H, *J* = 2.4 Hz), 7.86 (dd,1H, *J* = 8.5, 2.4 Hz). ^{13}C (100 MHz, $CDCl_3$) δ 27.7, 56.1, 83.0, 106.3, 115.6, 129.5, 134.5, 147.7, 150.5, 154.8. ESI HRMS: Found: *m/z* 369.1663. Calcd for $C_{17}H_{25}N_2O_7$: $(M+H)^+$ 369.1662.

Step 2: To a solution of iodobenzene (0.4 ml, 3.57 mmol) in THF (8 ml) cooled at –20 °C was added isopropylmagnesium chloride (1.8 ml, 2.0 M solution, 3.60 mmol) dropwise (Sapountzis and Knochel, 2002). The mixture was stirred at –20 °C under a N_2 atmosphere for 30 mins to allow I/Mg exchange. **B1** (553 mg, 1.5 mmol) was added and the mixture was left to be stirred at –20 °C for 2 h. Ethanol (2 mL) was then added to the reaction reaction mixture, followed by addition of $FeCl_2$ (0.38 g, 3 mmol), $NaBH_4$ (50 mg, 1.5 mmol) was added and the mixture was allowed to stir at room temperature under a N_2 atmosphere for 2 h. The reaction was finally quenched by water, and extracted thrice by diethyl ether. The combined organic extract was washed with brine, dried over anhydrous $MgSO_4$, filtered, and evaporated under reduced pressure to give the crude product. The crude product was purified by silica-gel flash column chromatography (*n*-hexane/ethyl acetate=70:30) to obtain the *N*-protected di-phenyl aniline (**B2**) (354 mg, 0.855 mmol, 57 %) as a pale brown solid.

^{1}H NMR (400 MHz, $CDCl_3$) δ 1.43 (s, 18H), 3.76 (s, 3H), 5.73 (br s, 1H), 6.59-6.62 (m, 2H), 6.93-6.97 (m, 2H), 7.08-7.10 (m, 2H), 7.28-7.30 (m, 2H). ^{13}C NMR (100 MHz, $CDCl_3$) δ 28.0, 55.5, 82.1, 101.1, 109.2, 118.2, 121.2, 121.5, 129.4, 129.5, 143.0, 144.0, 152.4, 155.3. ESI HRMS: Found: *m/z* 415.2236. Calcd for $C_{23}H_{31}N_2O_5$: $(M+H)^+$ 415.2233.

Step 3: To a stirred solution of acetyl chloride (41 μL, 0.579 mmol) in methanol (1 ml) was added a solution of **B2** (60 mg, 0.145 mmol) in methanol (0.4 ml) at 0 °C under a N_2 atmosphere. The mixture was then allowed to warm to room temperature before refluxed for 1 h. The reaction was then allowed to cool to room temperature and quenched by addition of saturated aqueous $NaHCO_3$, and extracted thrice with ethyl acetate. The combined organic extract was washed with brine, dried over anhydrous $MgSO_4$, filtered, and evaporated under

reduced pressure to give the crude product. The crude product was purified by silica-gel flash column chromatography (*n*-hexane/ethyl acetate=70:30) to obtain 3-methoxy-*N*-phenylbenzene-1,4-diamine (**B3**) in (24 mg, 0.113 mmol, 78%) as a dark purple solid.

^{1}H NMR (400 MHz, $CDCl_3$) δ 3.79 (s, 3H), 6.60 (dd, 1H, *J* = 2.4, 8.0 Hz), 6.65-6.68 (m, 2H), 6.79 (tt, 1H, *J* = 1.1, 7.3 Hz), 6.84-6.88 (m, 2H), 7.16-7.24 (m, 2H). ^{13}C NMR (100 MHz, $CDCl_3$) δ 55.7, 106.0, 114.8, 115.3, 115.7, 119.2, 129.5, 132.0, 134.1, 146.1, 148.1. ESI HRMS: Found: *m/z* 215.1186. Calcd for $C_{13}H_{15}N_2O$: $(M+H)^+$ 215.1184.

Step 4: To a solution of **B3** (21 mg, 0.100 mmol) in dichloromethane (1 ml) was added pyridine (13 μL, 0.16 mmol) and methyl 3-(chlorosulfonyl)thiophene-2-carboxylate (36 mg, 0.15 mmol) at room temperature under an inert atmosphere and left to be stirred for 3 h. The reaction was quenched by addition of water and extracted thrice with ethyl acetate. The combined organic extract was washed with brine, dried over anhydrous $MgSO_4$, filtered, and evaporated under reduced pressure to give the crude product. The crude product was purified by silica-gel flash column chromatography (*n*-hexane/ethyl acetate=60:40) to obtain GSK0660 (38 mg, 0.091 mmol, 91%) as an orange solid.

Methyl 3-(*N*-(2-methoxy-4-(phenylamino)phenyl)sulfamoyl)-thiophene-2-carboxylate (GSK0660) (Toth et al., 2012)

^{1}H NMR (400 MHz, $CDCl_3$) δ 3.52 (s, 3H), 4.01 (s, 3H), 5.65 (br s, 1H), 6.45 (d, 1H, *J* = 2.4 Hz), 6.58 (dd,1H, *J* = 2.4, 8.8 Hz), 6.94 (t, 1H, *J* = 7.2 Hz), 6.92-7.02 (m, 2H), 7.23-7.27 (m, 2H), 7.38-7.44 (m, 3H), 8.27 (br s, 1H). ^{13}C NMR (100 MHz, $CDCl_3$) δ 53.3, 55.6, 100.6, 109.9, 118.1, 118.4, 121.6, 126.9, 129.6, 129.7, 131.6, 131.8, 142.6, 142.8, 145.4, 152.8, 160.9. ESI HRMS: Found: *m/z* 419.0731. Calcd for $C_{19}H_{19}N_2O_5S_2$: $(M+H)^+$ 419.0735.

Synthesis of compound 10h (10h)

Compound 10h (IUPAC: methyl 3-(N-(4-(isopentylamino)-2-methoxyphenyl)sulfamoyl)-thiophene-2-carboxylate) was prepared according to reported synthetic route as shown in Scheme 3 (Toth et al., 2012).

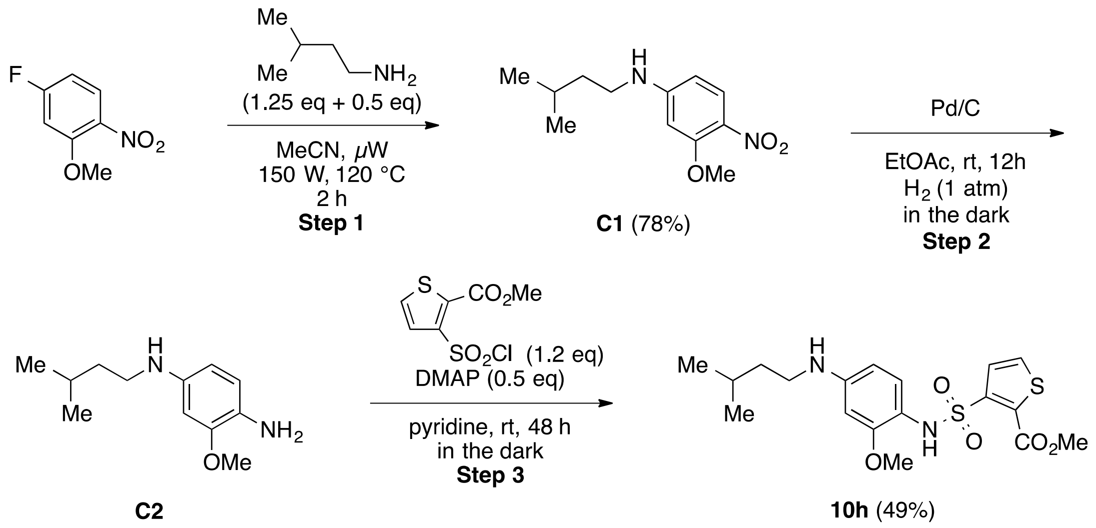

Scheme 3

Step 1: To a solution of 4-fluoro-2-methoxy-1-nitrobenzene (471 mg, 2.75 mmol) in anhydrous acetonitrile (2 ml) was added 3-methylbutan-1-amine (0.56 mL, 4.82 mmol) and the reaction mixture was heated to 120 °C under microwave irradiation in a sealed 10 mL reaction vessel for 2 h. The solvent was evaporated under reduced pressure, the residue was purified by silica-gel flash column chromatography (*n*-hexane/ethyl acetate=80:20) to obtain *N*-isopentyl-3-methoxy-4-nitroaniline (**C1**) (513 mg, 2.16 mmol, 78%) as a yellow solid. NMR analysis is in agreement with the literature data.

Step 2: To a solution of **C1** (460 mg, 1.93 mmol) in EtOAc (4 ml) was added Pd/C (63 mg, 10% Pd in carbon w/w%) under a N_2 atmosphere. The atmosphere was then removed in *vacuo* and replaced by hydrogen via balloon (1 atm). The reaction mixture was then left to be stirred at room temperature in the dark for 12 h. The catalyst was filtered off through a pad of celite and the filtrate was concentrated under reduced pressure to give the light sensitive aniline **C2**, which was directly used in the next step without further purification.

Step 3: Aniline **C2** was dissolved in anhydrous pyridine (2 mL) under a N_2 atmosphere. *N*,*N*-dimethylpyridin-4-amine (DMAP) (118 mg, 0.966 mmol) and methyl 3-(chlorosulfonyl)thiophene-2-carboxylate (558 mg, 2.32 mmol) were added and the reaction mixture was stirred for 48 h in the dark. The reaction was quenched by addition of water, and extracted thrice by dichloromethane. The combined organic extract was washed with brine, dried over anhydrous $MgSO_4$, filtered, and evaporated under reduced pressure to give the crude product. The crude product was purified by silica-gel flash column chromatography (*n*-

hexane/ethyl acetate=60:40) to obtain compound 10h (390 mg, 0.945 mmol, 49% in two steps) as a yellow solid.

Methyl 3-(*N*-(4-(isopentylamino)-2-methoxyphenyl)sulfamoyl)-thiophene-2-carboxylate (10h)

^{1}H NMR (400 MHz, $CDCl_3$) δ 0.92 (d, 6H, *J* = 6.8 Hz), 1.45 (q, 2H, *J* = 7.3 Hz), 1.67 (sept, 1H, *J* = 6.8 Hz), 3.04 (t, 2H, *J* = 7.4 Hz), 3.48 (s, 3H), 3.57 (br s, 1H), 3.99 (s, 3H), 5.92 (d, 1H, *J* = 2.4 Hz), 6.11 (dd, 1H, *J* = 8.6, 2.4 Hz), 7.27 (d, 1H, *J* = 8.6 Hz), 7.36 (s, 2H), 8.10 (br s, 1H); ^{13}C NMR (100 MHz, $CDCl_3$) δ 22.5, 25.9, 38.4, 42.1, 52.9, 55.1, 95.6, 104.4, 114.2, 128.1, 129.1, 131.4, 131.5, 145.6, 148.2, 153.4, 160.7.

^{1}H NMR Spectra of GW501516

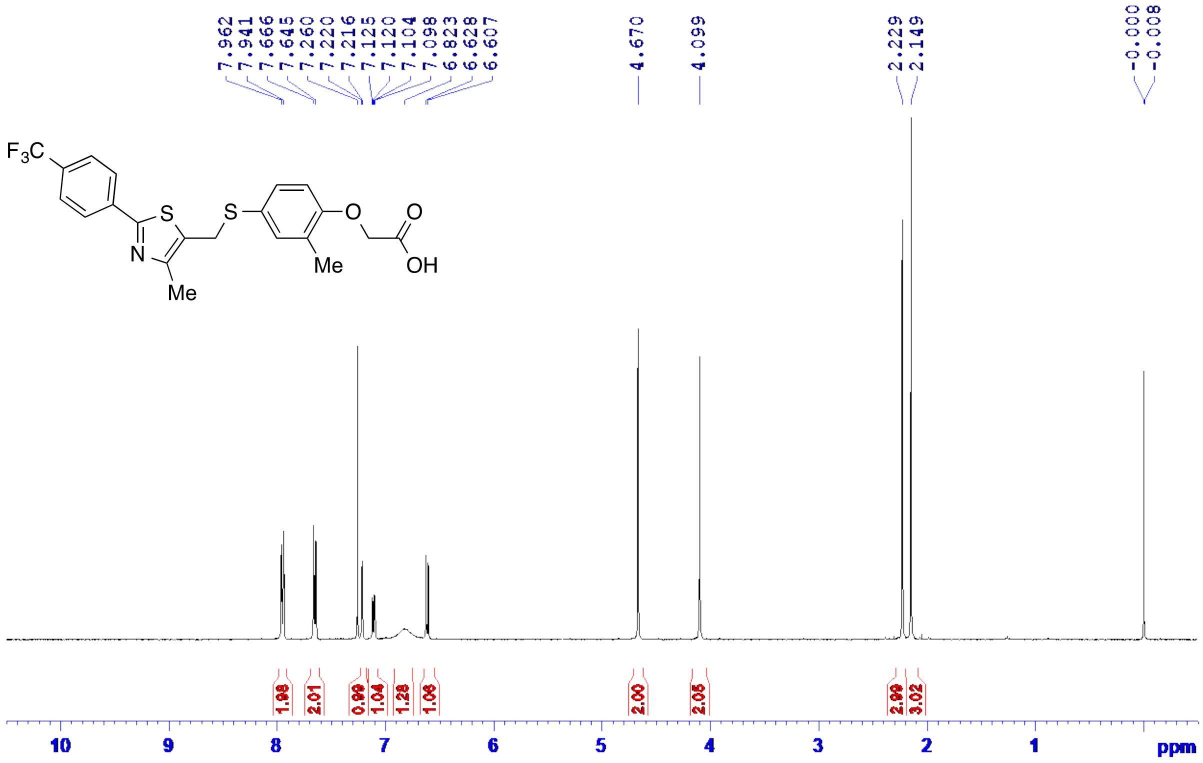

^{13}C NMR Spectra of GW501516

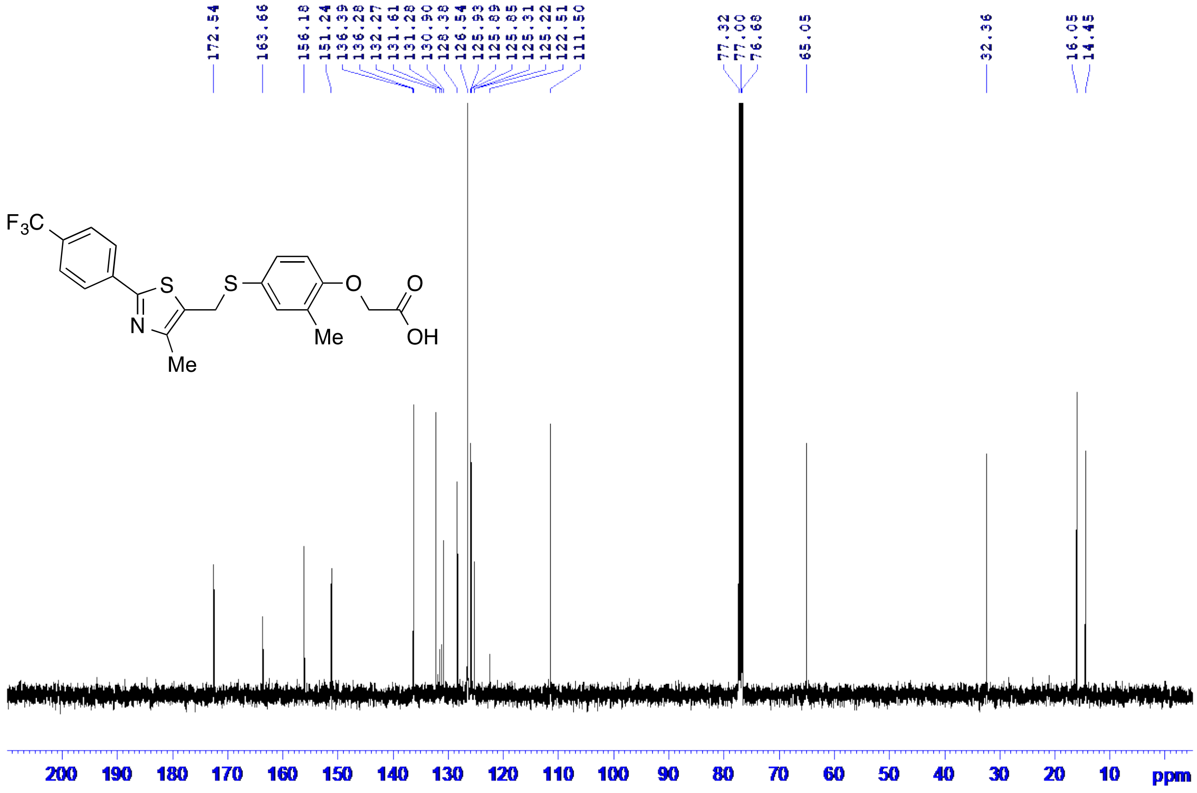

^{1}H NMR Spectra of GSK 0660

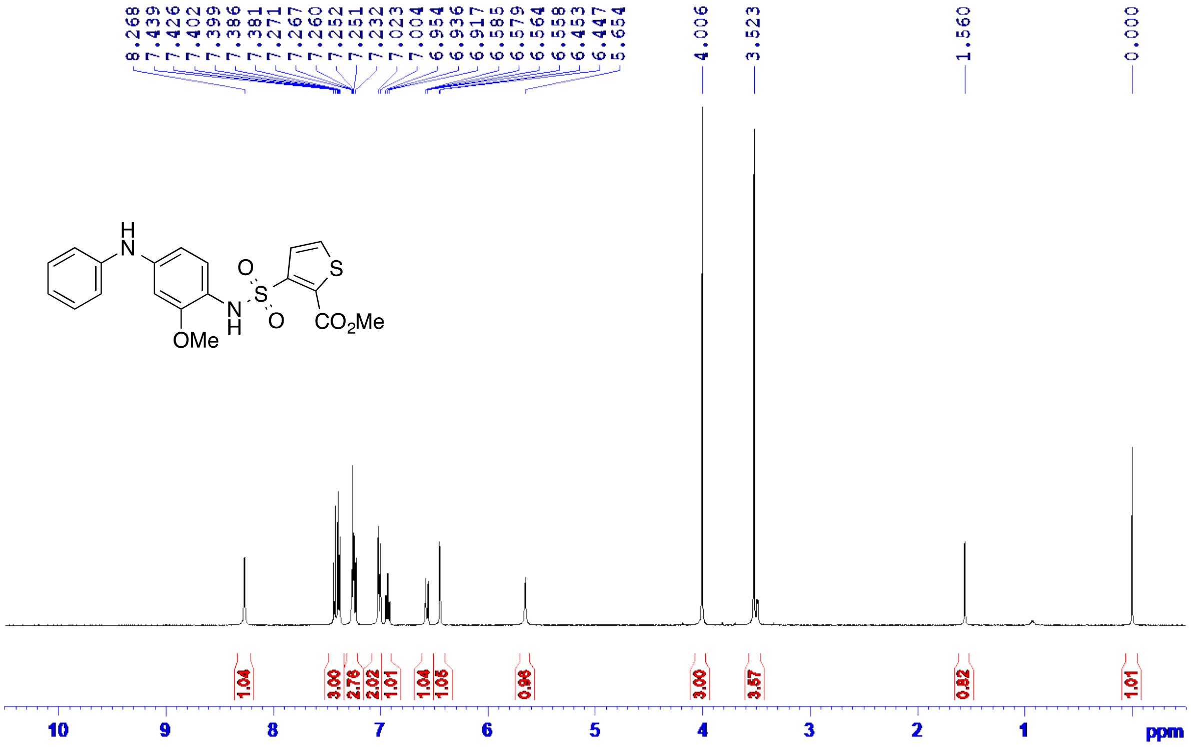

^{13}C NMR Spectra of GSK 0660

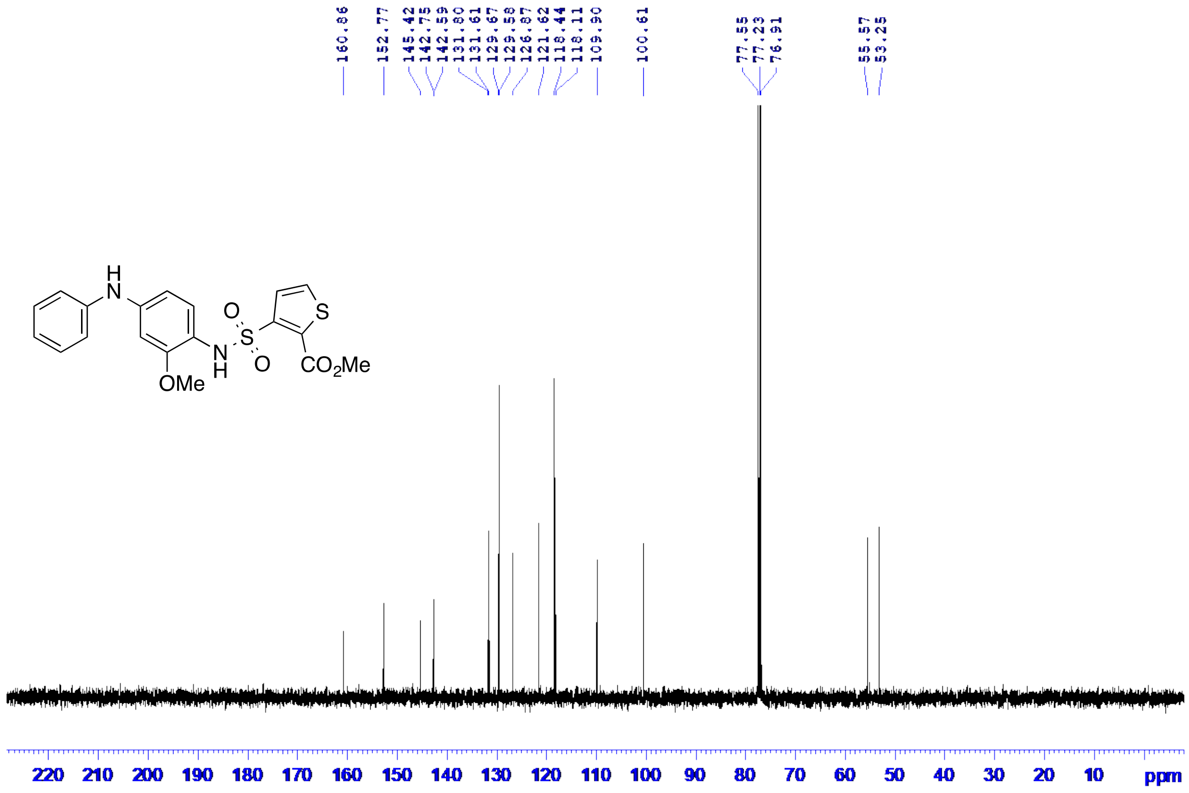

^{1}H NMR Spectra of 10h

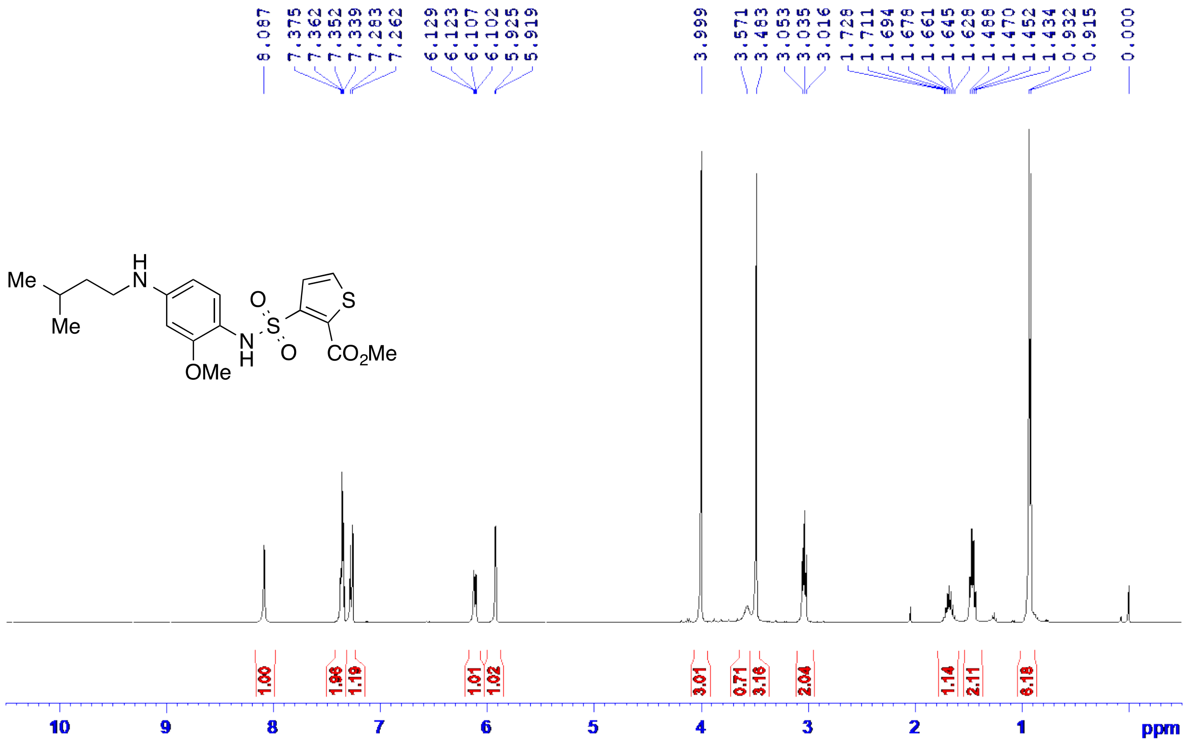

^{13}C NMR Spectra of 10h

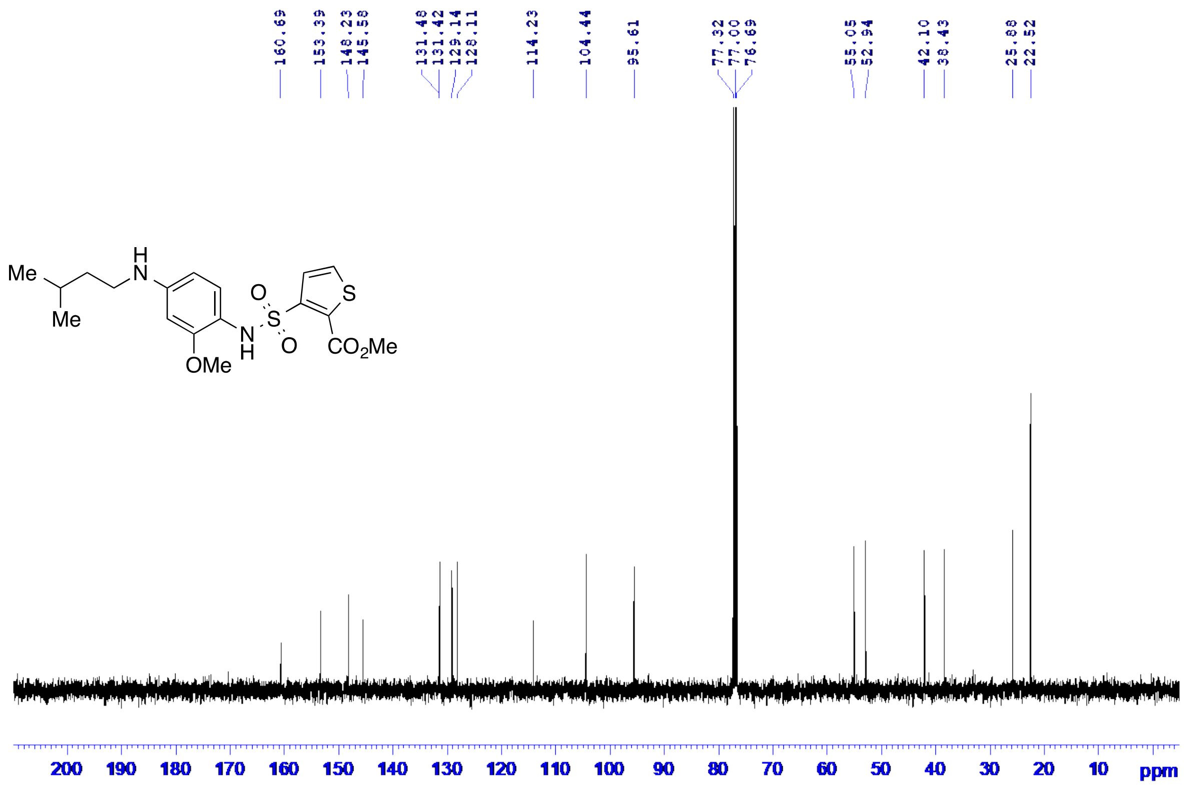

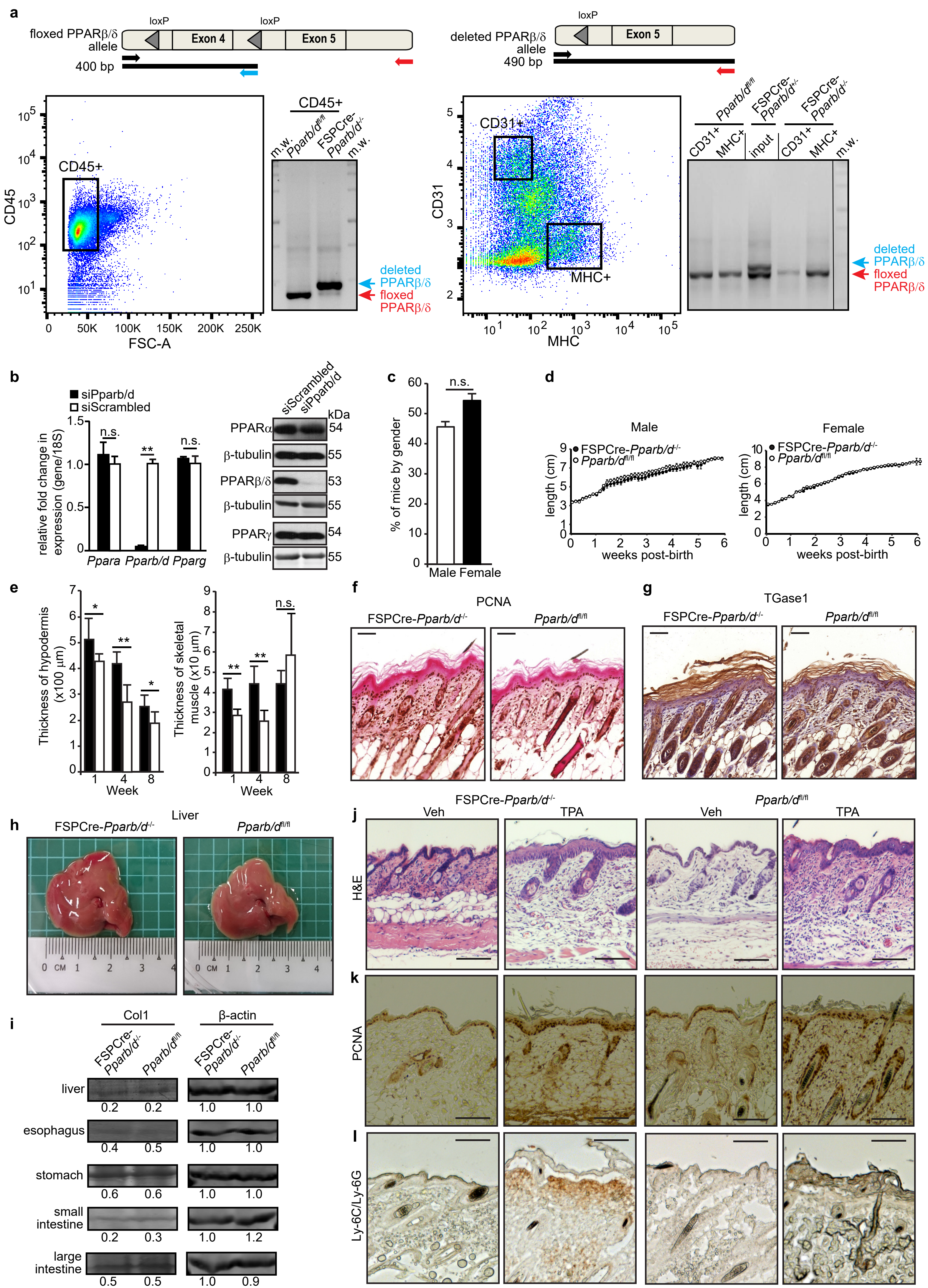
a
floxed PPARβ/δ allele
loxP
Exon 4
loxP
Exon 5
400 bp
deleted PPARβ/δ allele
490 bp
CD45
CD45+
FSC-A
m.w.
Ppparb/d fl/fl
FSPCre-Pparb/d-/-
deleted PPARβ/δ
floxed PPARβ/δ
CD31
CD31+
MHC+
MHC
input
b
siPparb/d
siScrambled
relative fold change in expression (gene/18S)
n.s.
**
Ppara Pparb/d Pparg
PPARα
β-tubulin
PPARβ/δ
PPARγ
kDa
54
55
53
c
% of mice by gender
Male Female
d
Male
Female
FSPCre-Pparb/d-/-
Pparb/d fl/fl
length (cm)
weeks post-birth
e
Thickness of hypodermis (x100 µm)
Thickness of skeletal muscle (x10 µm)
Week
f
PCNA
g
TGase1
h
Liver
i
Col1
β-actin
liver
esophagus
stomach
small intestine
large intestine
j
FSPCre-Pparb/d-/-
Pparb/d fl/fl
Veh
TPA
H&E
k
PCNA
l
Ly-6C/Ly-6G

Sng et al., Supplementary Figure S2

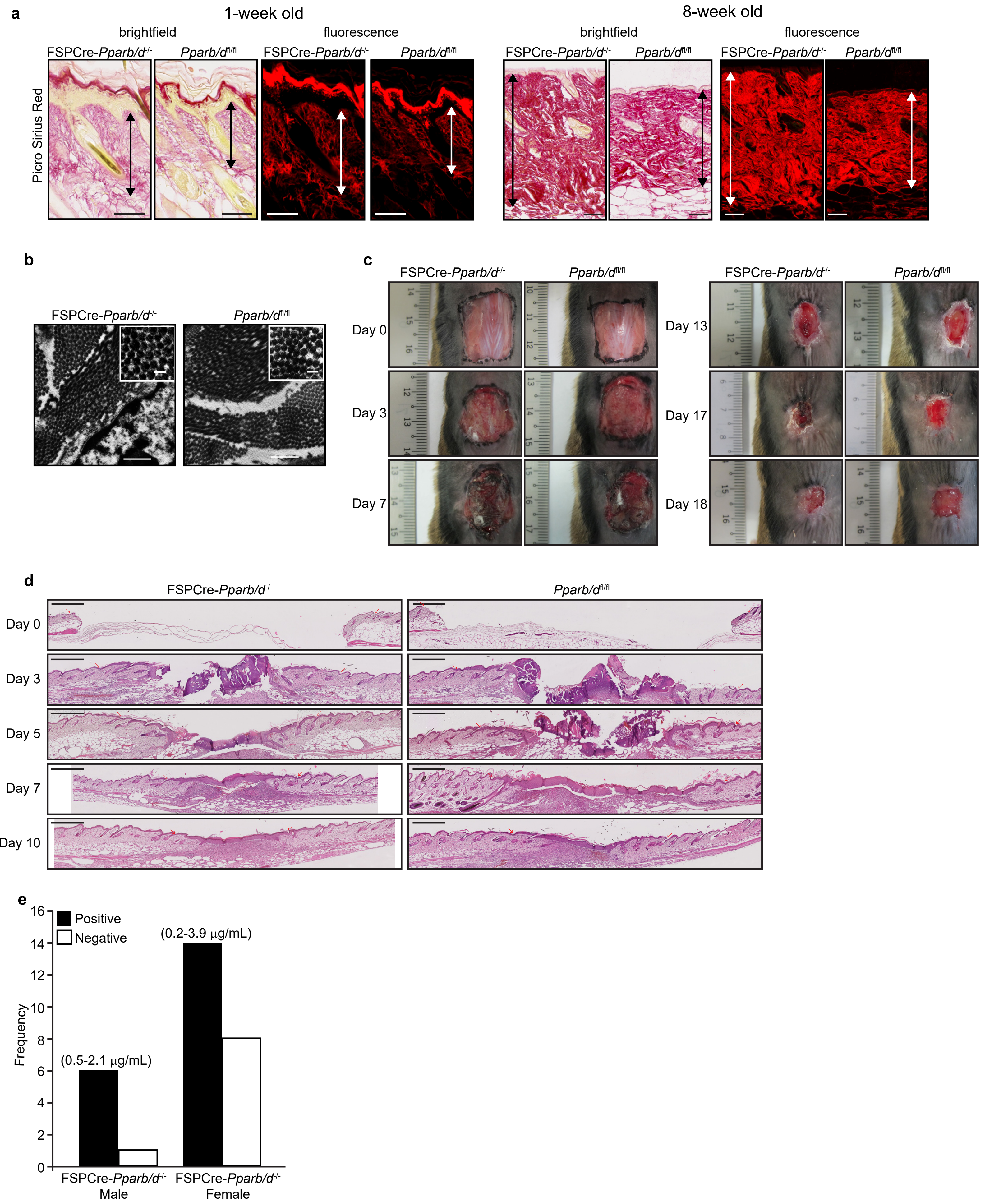

995 gene signature for human SSc

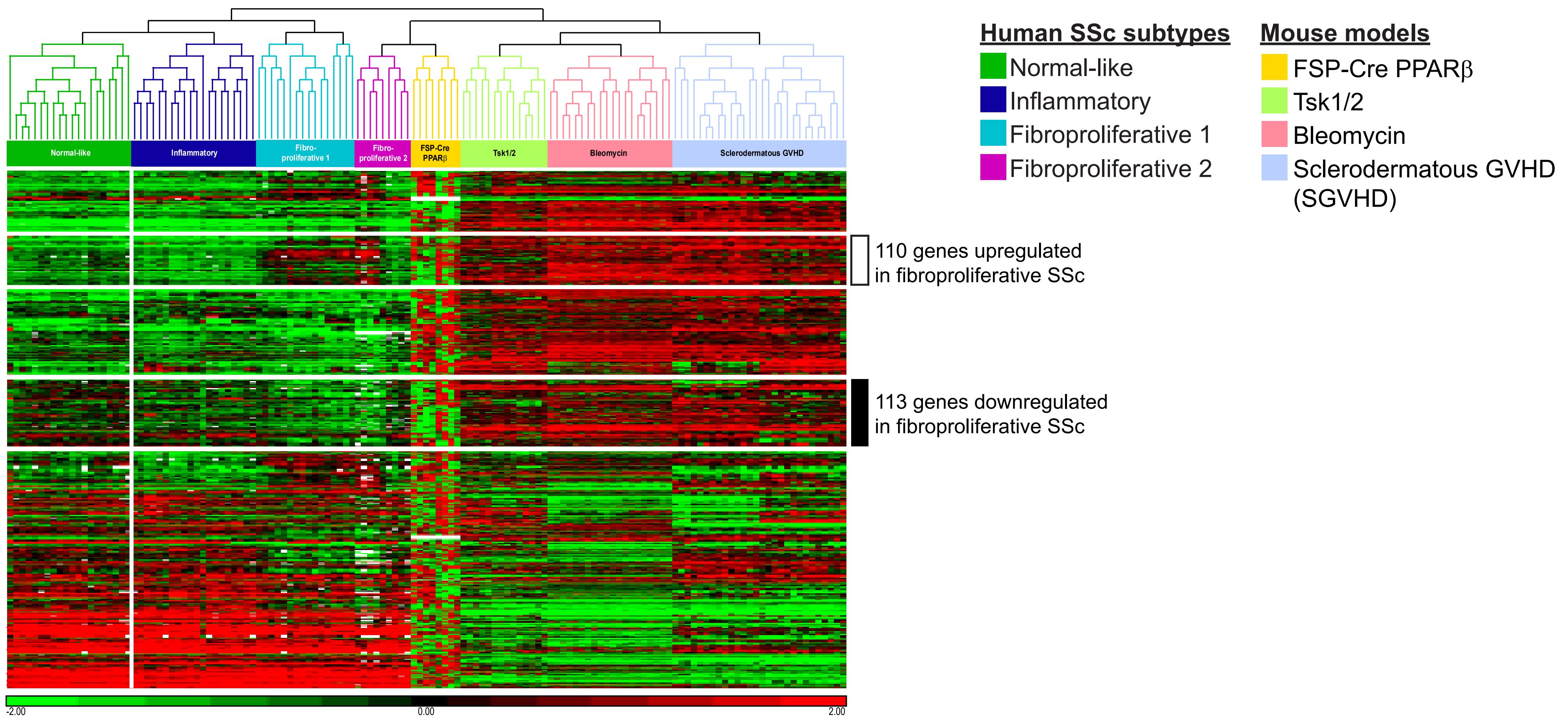

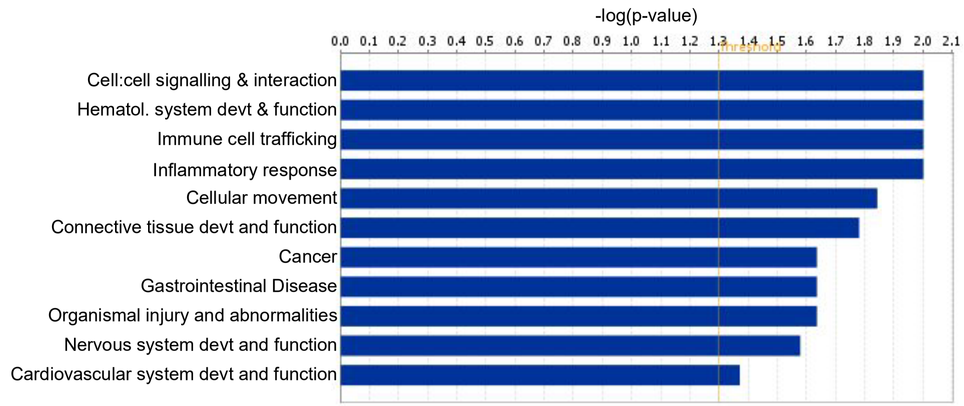

a

Epidermis

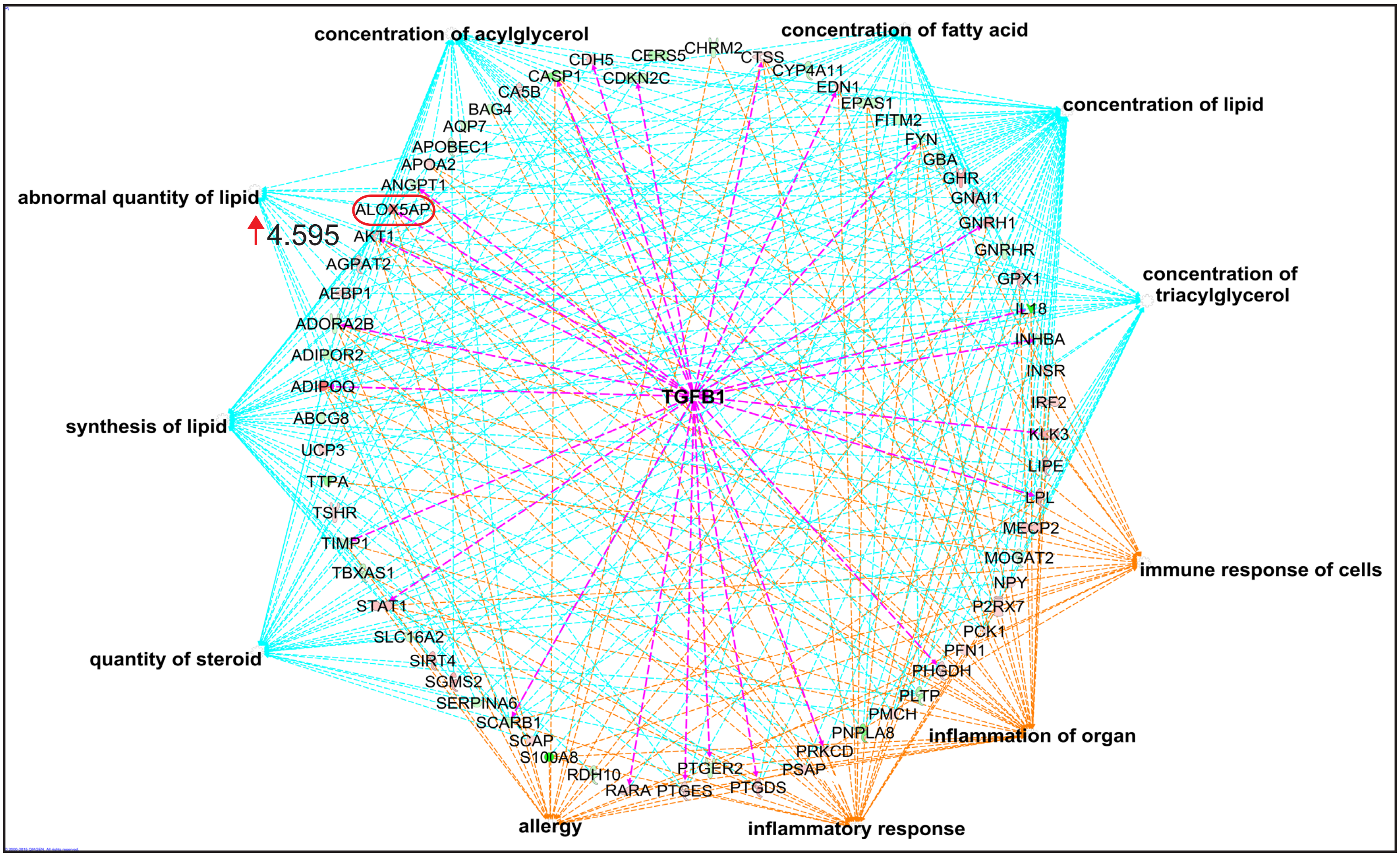

b

Dermis

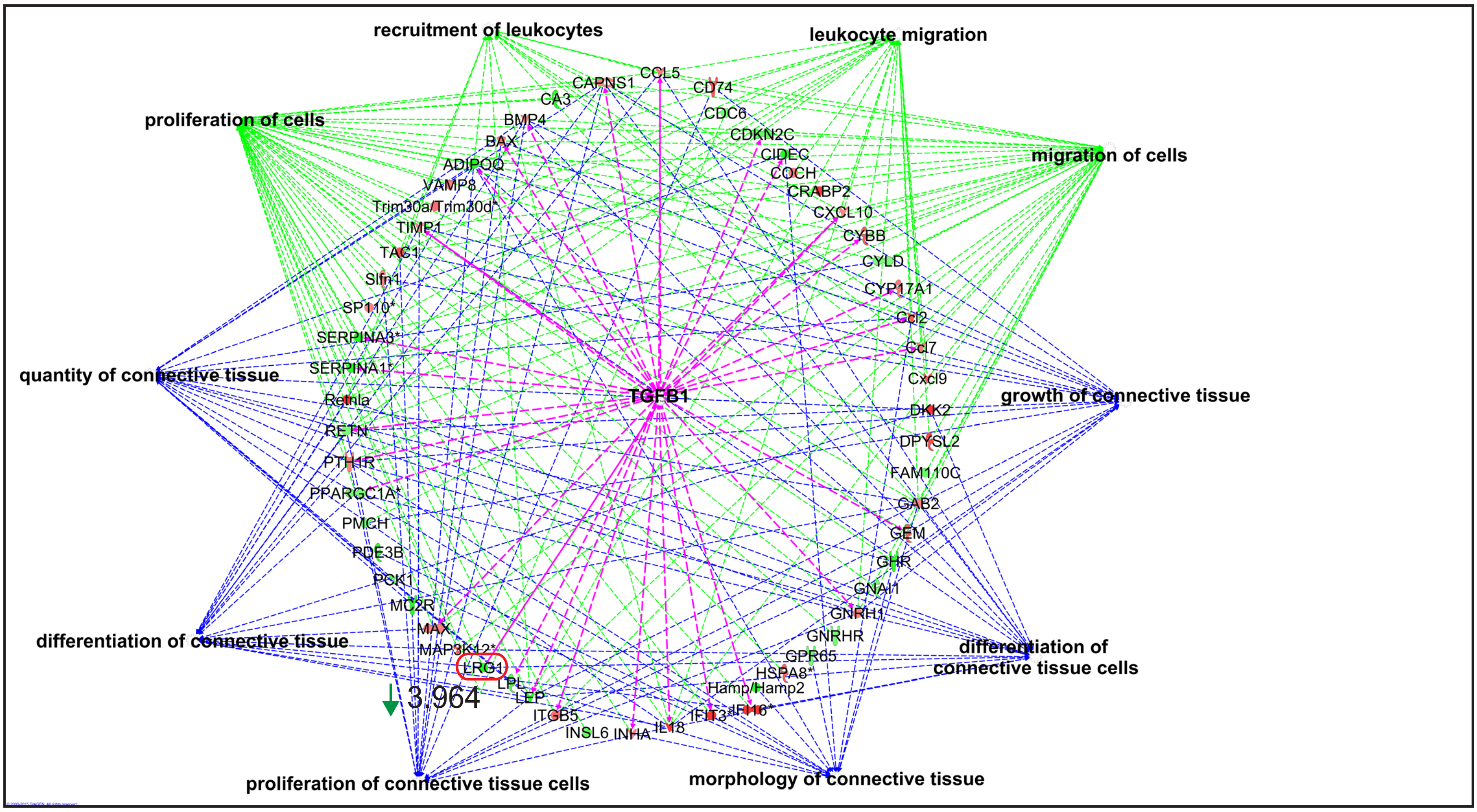

a

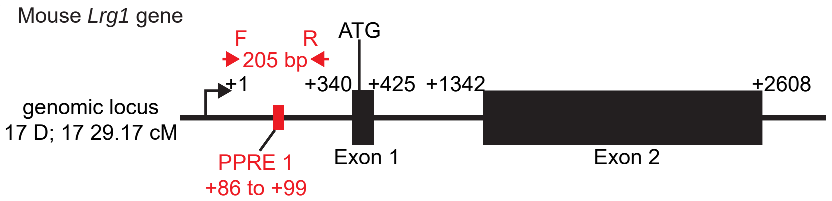

Designation	PPRE sequence	NUBIScan score
PPRE 1	AGGTGAcAGGAAA	0.719177

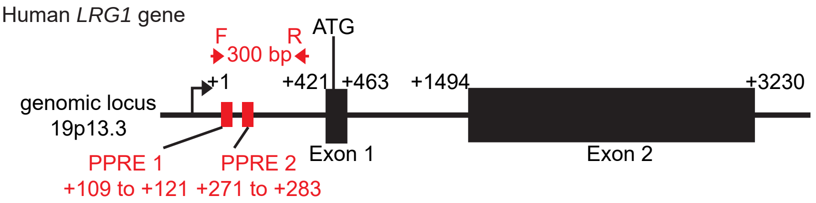

Designation	PPRE sequence	NUBIScan score
PPRE 1	AGTTCAaGGGTCA	0.789846
PPRE 2	AGGGGAgGGGTCA	0.732351

b

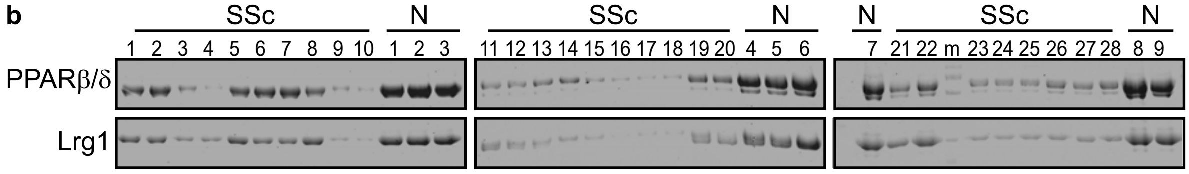

Supplement: Supplementary file 1 — Supplemental Information [file 41421_2018_14_MOESM1_ESM.pdf]
